# Supplementary material for: International epidemiology databases to evaluate AIDS (IeDEA) in sub-Saharan Africa, 2012–2019
Source: BMJ Open. 2020 May 15;10(5):e035246. doi: 10.1136/bmjopen-2019-035246 (PMC7232622; doi:10.1136/bmjopen-2019-035246)
Supplement: Author's manuscript [file bmjopen-2019-035246.draft_revisions.pdf]

BMJ Open

BMJ Open is committed to open peer review. As part of this commitment we make the peer review history of every article we publish publicly available.

When an article is published we post the peer reviewers' comments and the authors' responses online. We also post the versions of the paper that were used during peer review. These are the versions that the peer review comments apply to.

The versions of the paper that follow are the versions that were submitted during the peer review process. They are not the versions of record or the final published versions. They should not be cited or distributed as the published version of this manuscript.

BMJ Open is an open access journal and the full, final, typeset and author-corrected version of record of the manuscript is available on our site with no access controls, subscription charges or pay-per-view fees (http://bmjopen.bmj.com).

If you have any questions on BMJ Open's open peer review process please email info.bmjopen@bmj.com

Cohort profile: The International epidemiology Databases to Evaluate AIDS (IeDEA) in sub-Saharan Africa, 2012-2019

Journal:	*BMJ Open*
Manuscript ID	bmjopen-2019-035246
Article Type:	Cohort profile
Date Submitted by the Author:	24-Oct-2019
Complete List of Authors:	Chammartin, Frédérique; University of Bern, Institute of Social & Preventive Medicine<br>Dao Ostinelli, Cam Ha; University of Bern, Institute of Social & Preventive Medicine<br>Anastos, Kathryn; Albert Einstein College of Medicine, Department of Medicine<br>Jaquet, Antoine; University of Bordeaux, ISPED, Centre INSERM U1219 – Bordeaux Population Health<br>Brazier, Ellen ; City University of New York, Institute for Implementation Science in Population Health; City University of New York, Graduate School of Public Health and Health Policy<br>Brown, Steven; Indiana University Richard M Fairbanks School of Public Health, Department of Biostatistics<br>DABIS, FRANCOIS; University of Bordeaux, ISPED, Centre INSERM U1219 – Bordeaux Population Health<br>Davies, Mary-Ann; University of Cape Town, Centre for Infectious Disease Epidemiology and Research, School of Public Health and Family Medicine<br>Duda, Stephany N; Vanderbilt University School of Medicine, Department of Biomedical Informatics<br>Malateste, Karen; University of Bordeaux, ISPED, Centre INSERM U1219 – Bordeaux Population Health<br>Nash, Denis; City University of New York, Institute for Implementation Science in Population Health; City University of New York, Graduate School of Public Health and Health Policy<br>Wools-Kaloustian, Kara; Indiana University School of Medicine, Department of Medicine<br>von Groote, Per M; University of Bern, Institute of Social & Preventive Medicine<br>Egger, Matthias; University of Bern, Institute of Social & Preventive Medicine
Keywords:	HIV & AIDS < INFECTIOUS DISEASES, EPIDEMIOLOGY, INFECTIOUS DISEASES, Tuberculosis < INFECTIOUS DISEASES

I, the Submitting Author has the right to grant and does grant on behalf of all authors of the Work (as defined in the below author licence), an exclusive licence and/or a non-exclusive licence for contributions from authors who are: i) UK Crown employees; ii) where BMJ has agreed a CC-BY licence shall apply, and/or iii) in accordance with the terms applicable for US Federal Government officers or employees acting as part of their official duties; on a worldwide, perpetual, irrevocable, royalty-free basis to BMJ Publishing Group Ltd ("BMJ") its licensees and where the relevant Journal is co-owned by BMJ to the co-owners of the Journal, to publish the Work in this journal and any other BMJ products and to exploit all rights, as set out in our licence.

The Submitting Author accepts and understands that any supply made under these terms is made by BMJ to the Submitting Author unless you are acting as an employee on behalf of your employer or a postgraduate student of an affiliated institution which is paying any applicable article publishing charge ("APC") for Open Access articles. Where the Submitting Author wishes to make the Work available on an Open Access basis (and intends to pay the relevant APC), the terms of reuse of such Open Access shall be governed by a Creative Commons licence – details of these licences and which Creative Commons licence will apply to this Work are set out in our licence referred to above.

Other than as permitted in any relevant BMJ Author's Self Archiving Policies, I confirm this Work has not been accepted for publication elsewhere, is not being considered for publication elsewhere and does not duplicate material already published. I confirm all authors consent to publication of this Work and authorise the granting of this licence.

For peer review only

Version 4. Target journal: BMJ Open

Cohort profile: The International epidemiology Databases to Evaluate AIDS (IeDEA) in sub-Saharan Africa, 2012-2019

Frédérique Chammartin[1], Cam Ha Dao Ostinelli[1], Kathryn Anastos[2], Antoine Jaquet,[3] Ellen Brazier[4,5], Steven Brown[6], François Dabis[3], Mary-Ann Davies[7], Stephany N Duda[8], Karen Malateste[3], Denis Nash[4,5], Kara K Wools-Kaloustian[9], Per M von Groote[1], Matthias Egger[1,7]

[1] Institute of Social and Preventive Medicine, University of Bern, Bern, Switzerland
[2] Department of Medicine, Albert Einstein College of Medicine, Bronx, NY, USA
[3] Université Bordeaux, ISPED, Centre INSERM U1219 – Bordeaux Population Health, Bordeaux, France
[4] Institute for Implementation Science in Population Health, City University of New York, NY, USA
[5] Graduate School of Public Health and Health Policy, City University of New York, NY, USA
[6] Department of Biostatistics, Indiana University Fairbanks School of Public Health, Indianapolis, IN, United States
[7] Centre for Infectious Disease Epidemiology and Research, School of Public Health and Family Medicine, University of Cape Town, South Africa
[8] Department of Biomedical Informatics, Vanderbilt School of Medicine, Nashville, TN, USA
[9] Department of Medicine, Indiana University School of Medicine, Indianapolis, IN, United States

Correspondence to:

Professor Matthias Egger
Institute of Social and Preventive Medicine (ISPM)
University of Bern
Mittelstrasse 43
CH-3012 Bern
Switzerland
matthias.egger@ispm.unibe.ch

Abstract 293 words, main text 2426 words, 2 tables, 3 figures, 1 Box with strengths and weaknesses (227 words), footnotes (375 words), 69 references

For peer review only

ABSTRACT

Purpose: The objectives of the International epidemiology Databases to Evaluate AIDS (IeDEA) are to (i) evaluate the delivery of combination antiretroviral therapy (ART) in children, adolescents and adults in sub-Saharan Africa, (ii) to describe ART regimen effectiveness, durability and tolerability, (iii) to examine HIV-related comorbidities and co-infections, and (iv) to examine the pregnancy- and HIV-related outcomes of women on ART and their infants exposed to HIV or antiretroviral therapy in utero or via breastmilk.

Participants: IeDEA is organized in four regions (Central, East, Southern and West Africa), with 240 treatment and care sites, six data centres at African, European and US universities, and almost 1.4 million children, adolescents and adult people living with HIV (PLWHIV) enrolled.

Findings to date: The data include socio-demographic characteristics, clinical outcomes, opportunistic events, treatment regimens, clinic visits and laboratory measurements. They have been used to analyse outcomes in people living with HIV-1 or HIV-2 who initiate ART, including determinants of mortality, of switching to second-line and third-line ART, drug resistance, loss to follow-up and the immunological and virological response to different ART regimens. Programme-level estimates of mortality have been corrected for loss to follow-up. We examined the impact of co-infection with hepatitis B and C, and the epidemiology of different cancers and of (multi-drug resistant) tuberculosis, renal disease and of mental illness. The adoption of "Treat All", making ART available to all PLWHIV regardless of $CD4^+$ cell count or clinical stage was another important research topic.

Future plans: IeDEA has formulated several research priorities for the "Treat All" era in sub-Saharan Africa. It recently obtained funding to set up sentinel sites where additional data are prospectively collected on cardiometabolic risks factors as well as mental health and liver diseases, and is planning to create a drug resistance database.

For peer review only - http://bmjopen.bmj.com/site/about/guidelines.xhtml

INTRODUCTION

The introduction of combination antiretroviral therapy (ART) in sub-Saharan Africa from 2004 onwards has substantially improved the prognosis of HIV-1 infection, with a decline in AIDS-related deaths [1] and a decline in the incidence of new HIV-1 infections [2]. However, in many settings HIV/AIDS is still a public health threat. An estimated 1.8 million new infections occurred in 2017 and almost a million adult and child deaths were due to HIV, most of them in sub-Saharan Africa [2].

The World Health Organization (WHO), the Joint United Nations Programme on HIV/AIDS (UNAIDS) and many of the countries most heavily affected by the HIV epidemic have committed to ending HIV/AIDS as a major public health problem by 2030.[1] Targets to be reached by 2020 include that 90% of people living with HIV (PLWHIV) be aware of their status, 90% of those diagnosed initiate ART, and 90% of those on ART achieve undetectable viral loads (the 90-90-90 targets) [3]. Progress towards these goals has been substantial, especially in the most affected regions of Eastern and Southern Africa. The latest estimates suggested that the two regions are on track and had achieved 76%, 79% and 83% of the 90-90-90 targets, respectively in 2017 [4]. West and Central Africa face difficulties with the first 90% target and only an estimated 42% of PLWHIV in these regions were aware of their status [4].

Established more than ten years ago by the National Institutes of Health (NIH), the International epidemiology Databases to Evaluate AIDS (IeDEA) are a global cohort collaboration that collects HIV/AIDS data from HIV care and treatment programs, including in sub-Saharan Africa. The regional IeDEA data centres consolidate, curate and analyse data to evaluate the outcomes of people living with HIV/AIDS and monitor progress. The first years of the cohorts in sub-Saharan Africa were described previously [5]; here we provide an update on methods, key data and future plans.

COHORT DESCRIPTION

In 2006 the National Institute of Allergy and Infectious Diseases (NIAID) sought applications for a global consortium structured through regional centres to pool clinical and epidemiological data on PLWHIV, in order to address questions that could not be answered by individual cohorts [5]. IeDEA covers seven geographic regions, namely

For peer review only - http://bmjopen.bmj.com/site/about/guidelines.xhtml

North America, the Caribbean and Central/South America, the Asia-Pacific and four regions in sub-Saharan Africa: West Africa, Central Africa, East Africa and Southern Africa. The project was initially funded for a 5-year period and has since been extended twice, with the current funding cycle ending in 2021.

Settings and number of PLWHIV enrolled

To date, the African regions of IeDEA received data from 240 HIV care and treatment facilities in 19 sub-Saharan African countries (Figure 1A). Close to 1,400,000 PLWHIV who initiated ART in sub-Saharan Africa are included (Figure 1B), of whom over 680,000 are currently in care. In East and Southern Africa, both urban and rural facilities are well represented, while in Central and West Africa, urban facilities dominate. Facilities are predominately public (95%) and operated at the primary or secondary care level, with the exception of West Africa where 70% of facilities are at the tertiary level of care (Table 1).

Data collection at individual and site level

Since its inception, IeDEA has collected routine clinical data of PLWHIV followed under treatment, which includes socio-demographic characteristics, clinical outcomes, opportunistic events, treatment regimens, clinic visits and laboratory measurements. More recently, the IeDEA network has developed a Data Exchange Standard (DES) protocol (see www.iedeades.org) with 25 data tables, which include a total of 228 unique variables (36 compulsory and 192 additional variables) and within-region unique patient research identifiers. Standardized data collection is supported by eight codebooks, including the Anatomical Therapeutic Chemical (ATC) classification for drugs and lists with codes for reasons for stopping treatment, for dropping out of the cohort, for mode of HIV infection, country, type and site of comorbidities, laboratory measurements and units of measurements and type of viral load assay. The collection, management and sharing of data is facilitated by the Harmonist toolkit, a software and standards package that supports research projects through the Research Electronic Data Capture (REDCap) system [6].

In recent years, site assessments and site surveys have been conducted on a regular basis to collect up-to-date information related to available clinical service and care models in the participating facilities. For example, a study compared the characteristics and

For peer review only - http://bmjopen.bmj.com/site/about/guidelines.xhtml

comprehensiveness of adult HIV care and treatment programmes in sub-Saharan Africa with programmes in the Americas and Asia-Pacific region [7]. Other studies examined the management of mental health and substance use disorders [8], or the diagnostic and screening practices for (drug resistant) tuberculosis in adult and paediatric patients [9–11]. Furthermore, the routine data collected by participating sites have been enriched in some countries by linking the routine HIV databases to cancer registries [12,13], vital registries [14] or administrative databases [15].

Trends in CD4 cell count and viral load measurements

While WHO continues to recommend a CD4 cell count before starting ART to inform the management of advanced disease and differentiated care in the Treat All era, it also recommends that CD4 testing be replaced by viral load measurement for monitoring of treatment and identification of treatment failure [16]. Figure 2 shows that in Southern and West Africa, the number of CD4 measurements tended to be stable over time, despite an increasing number of PLWHIV in care, while in East and Central Africa the number of CD4 measurements dropped. At present, 53% of the active facilities reported routine CD4 testing and 65% routine viral load testing (Table 1). The United States President's Emergency Plan for AIDS Relief (PEPFAR), which provides substantial funding for AIDS treatment, care and prevention in countries most affected by the epidemic, has progressively reduced its support for CD4 testing [17].

Trends in antiretroviral therapy

Until recently, the recommended first-line ART regimen in sub-Saharan Africa consisted of two nucleoside reverse transcriptase inhibitors and one non-nucleoside reverse transcriptase inhibitor (2NRTIs+1NNRTI). The combination of tenofovir (TDF), lamivudine (3TC) (or emtricitabine (FTC)) and efavirenz (EFV) is the current treatment of choice. The phasing out of stavudine (D4T) and nevirapine (NVP) was almost complete in 2014 (Table 2). East Africa and Southern Africa are currently rolling out dolutegravir (DTG), an integrase inhibitor with a high barrier to resistance [18][19]. Due to concerns about an increased risk of neural tube defects if taken during pregnancy[20], the roll-out to women has been delayed or limited in some settings. Of note, drug stock-outs in the last 12

For peer review only - http://bmjopen.bmj.com/site/about/guidelines.xhtml

months were reported by 59 facilities for first-line drugs, and by 45 for second-line drugs (Table 1).

Mortality and retention in care

In cohorts of PLWHIV who initiated ART in consecutive two-year periods from 2001 to 2016, mortality at 3 years declined substantially in all African IeDEA regions (Figure 3A). Loss to follow-up, defined as more than 90 days late to the next scheduled visit, remained substantial in all regions, and particularly high in Southern Africa (Figure 3B). Retention in care is key to the success of the public health approach to ART. Loss to follow-up has been an important issue for IeDEA, and activities to trace PLWHIV not returning to the clinic have increased in recent years. At present, tracing of PLWHIV on ART who were lost to follow-up is in place in 89% of the active facilities; 75% have implemented it routinely (Table 1). Tracing methods vary widely across facilities and include phone calls and home visits by clinic staff or community health workers.

Patient and public involvement

IeDEA is based on the collection of routine clinical data and no patients were involved in developing the research question, outcome measures and overall design of the collaboration. Due to the anonymous nature of the data, we cannot disseminate the results of analyses of the data directly to study participants.

KEY RESEARCH AREAS AND PUBLICATIONS

Over 500 publications in MEDLINE acknowledge funding from a core grant from the NIH to one or several African IeDEA regions, and these publications have been cited over 10,000 times. Multiregional projects are developed in IeDEA working groups, which currently address eight clinical areas: (i) cancer, (ii) ART outcomes, (iii) hepatitis, (iv) mental health, (v) mother-infant and paediatrics, (vi) renal disease, (vii) substance use and (viii) tuberculosis. Multiregional research concepts are discussed in the Executive Committee of IeDEA, revised and approved or rejected. In recent years, several analyses were done in collaboration with WHO or UNAIDS [21–23]. The number of publications reporting multi-regional analyses from several African IeDEA regions increased over time, from one such publication in 2007 to 24 multiregional publications in 2018. Some of the

For peer review only - http://bmjopen.bmj.com/site/about/guidelines.xhtml

key studies are summarised below, with a focus on more recent and on multiregional analyses.

Treatment outcomes in adults, adolescents, children and pregnant women: Several studies examined outcomes in people living with HIV-1 or HIV-2 who initiate ART, including determinants of mortality, of switching to second-line and third-line ART, drug resistance, loss to follow-up and the immunological and virological response to different ART regimens [24–37]. For example, the African IeDEA regions contributed importantly to a large-scale analysis of outcomes in adolescents living with perinatally acquired HIV, which showed that HIV-associated mortality during adolescence was substantially higher in sub-Saharan Africa, South and Southeast Asia, and South America and the Caribbean than in Europe [36]. A similar analysis of adolescents living with HIV showed that mortality and loss to follow-up were worse among those entering care at 15 years or older [37]. The authors concluded that adolescents must be evaluated separately from younger children and adults to identify population-specific reasons for death and loss to follow-up [37].

Programme-level mortality: It became clear early on during the scale-up of ART in sub-Saharan Africa, that loss to follow-up of patients initiating ART was substantial [38], and that mortality among patients lost was higher than among patients remaining in care [39]. Ignoring loss to follow-up might thus bias programme-level estimates of mortality, and much effort has gone into correcting programme-level mortality for loss to follow-up [40–46]. For example, an analysis of all four African regions showed that when analysing the uncorrected data observed in the clinics, 52% of adults and children were retained on ART, 42% were lost to follow-up and 6% had died 5 years after ART initiation [46]. After accounting for undocumented deaths and self-transfers, an estimated 67% of patients were retained on ART, 19% had stopped ART and 15% had died [46].

Co-infections and co-morbidities: IeDEA investigators have examined the prevalence and impact of co-infection with hepatitis B and C, and the epidemiology of different cancers and of (multi-drug resistant) tuberculosis, renal disease and of mental illness [8,47–58]. For example, an analysis of IeDEA data and data from the Collaboration of Observational HIV Epidemiological Research in Europe (COHERE) [59] showed that children living with HIV from sub-Saharan Africa, but not those from Europe or Asia had a high risk of developing Kaposi sarcoma after starting ART [58]. Similarly, a recent analysis

For peer review only - http://bmjopen.bmj.com/site/about/guidelines.xhtml

of IeDEA and COHERE data showed that compared to European women, rates of cervical cancer were 11 times higher in South Africa [50]. A recent multiregional study of multi-drug resistant tuberculosis included HIV positive and HIV negative adults with tuberculosis from seven high-burden countries (Côte d'Ivoire, Democratic Republic of the Congo, Kenya, Nigeria, South Africa, Peru, and Thailand). Molecular or phenotypic drug susceptibility testing was done locally and at a reference laboratory. The results showed that inaccurate local drug susceptibility testing led to under-treatment of drug-resistant tuberculosis and increased mortality [54].

The challenge of "Treat All": Nearly all countries in sub-Saharan Africa have now adopted national polices to offer ART to all PLWHIV regardless of CD4 cell count or clinical stage ('Treat All'), in order to meet the UNAIDS 90-90-90 targets. In 2011, Malawi was one of the first countries to implement such a strategy for the prevention of mother-to-child transmission (PMTCT), recommending ART for pregnant and breastfeeding women living with HIV, regardless of CD4 cell count or WHO clinical stage ("Option B+"). An IeDEA analysis of the Malawian experience showed that poor retention in care was a problem in many facilities, with early loss to follow-up particularly high in facilities with a high patient volume and in patients who start ART during pregnancy on the day of HIV diagnosis [35]. More recently, IeDEA investigators used regression discontinuity analysis to examine changes in rapid HIV treatment initiation after national "Treat All" policy adoption in six countries (Burundi, Kenya, Malawi, Rwanda, Uganda, Zambia) [60]. They showed a strong and sustained effect of the adoption of "Treat All" policies on ART initiation within 30 days of enrollment in HIV care in all six countries [60].

THE FUTURE

At the end of 2018, IeDEA published a consensus statement [61] and a journal supplement [62] on research priorities to inform the implementation of the "Treat All" policy in children and adolescents[63], pregnant and post-partum women [64], and for mental health, substance use [65] and drug resistance [66]. These documents will guide IeDEA's future research agenda in sub-Saharan Africa. Furthermore, the creation of an IeDEA Sentinel Research Network (IeDEA-SRN) will facilitate the collection of detailed data in selected IeDEA sites on cardio-metabolic risk factors (e.g. hypertension, diabetes,

For peer review only - http://bmjopen.bmj.com/site/about/guidelines.xhtml

dyslipidaemia) liver disease (liver fibrosis and steatosis) , mental health and substance use. In the East and Southern African regions, pharmacovigilance in pregnancy is being developed to assess the impact of ART on birth outcomes. A project involving all four African regions studies the cascade of screening for cervical cancer, while the establishment of the South African HIV Cancer Match (SAM) study of over ten million PLWHIV, from linkage of national laboratory with cancer registry data, will allow the study of less common cancers. Finally, the creation of a drug resistance database as a central repository for resistance tests performed in routine clinical care is another planned addition.

COLLABORATIONS

The African regions of IeDEA have collaborated and continue to encourage collaborations with other consortia, cohort collaborations, the HIV modelling community and public health agencies as well as individuals wishing to use IeDEA data. Examples include work with COHERE [50,58,67,68], the Collaborative Initiative for Paediatric HIV Education and Research (CIPHER) collaboration [36,69] or the Measurement & Surveillance of HIV Epidemics (MeSH) consortium [22] as well as UNAIDS [22,23] and WHO [21]. Furher collaborations are welcome. Investigators wishing to work with the IeDEA data should contact the teams at the regional data centres (see www.iedea.org for contact details) and send a concept sheet for the analyses they are interested in performing and the variables that would be required. Anyone wishing to work with IeDEA must sign a data-use agreement.

For peer review only

For peer review only - http://bmjopen.bmj.com/site/about/guidelines.xhtml

FOOTNOTES

Acknowledgements: The authors wish to thank the clinical and administrative staff at the participating clinics. We are grateful to all PLWHIV who contributed to the IeDEA database.

Contributors: FC, PMG, and ME conceptualised the study. FC and CHDO performed statistical analyses. FC and ME wrote the first draft of the paper. CHDO, KA, AJ, EB, SB, FD, MAD, SND, KM, BSM, DN, KWK, CY, PMG and ME contributed to interpreting the data and to the writing and revising of the manuscript.

Funding: The International Epidemiology Databases to Evaluate AIDS (IeDEA) is supported by the U.S. National Institutes of Health's National Institute of Allergy and Infectious Diseases, the Eunice Kennedy Shriver National Institute of Child Health and Human Development, the National Cancer Institute, the National Institute of Mental Health, the National Institute on Drug Abuse and Alcoholism, the National Institute of Diabetes and Digestive and Kidney Diseases, the Fogarty International Center, the National Library of Medicine, and the Office of the Director: Central Africa, U01AI096299; East Africa, U01AI069911; Southern Africa, U01AI069924; West Africa, U01AI069919. Informatics resources are supported by the Harmonist project, R24AI124872. ME was supported by special project funding (Grant No. 174281) from the Swiss National Science Foundation.

Disclaimer: The contents are the responsibility of the authors and do not necessarily reflect the views of NIAD or the US Government. The funders had no role in study design, data collection and analysis, decision to publish or preparation of the manuscript.

Competing interests: None declared.

Ethics approval: Ethics Committee of the Canton of Bern, Switzerland.

Provenance and peer review: Not commissioned; externally peer reviewed.

Data sharing statement: Investigators wishing to work with IeDEA data should contact the regional data centres (see www.iedea.org) and send a concept sheet for the analyses they are interested in performing and the variables that would be required. See

For peer review only - http://bmjopen.bmj.com/site/about/guidelines.xhtml

www.iedeades.org for list of variables. Those wishing to work with the data must sign a data-use agreement.

Open Access: This is an Open Access article distributed in accordance with the Creative Commons Attribution Non Commercial (CC BY-NC 4.0) license, which permits others to distribute, remix, adapt, build upon this work non-commercially, and license their derivative works on different terms, provided the original work is properly cited and the use is non-commercial. See: http://creativecommons.org/licenses/by-nc/4.0/.

For peer review only

Box: Strengths and limitations of this study

- An important strength of the IeDEA cohort collaboration in sub-Saharan Africa is its large size, which allows analyses of outcomes of antiretroviral therapy (ART) in children, adolescents and pregnant and postpartum women, across diverse settings.
- The data reflect routine care across a wide range of real-world settings during the scale up of ART in sub-Saharan Africa and thus provide a valuable platform to conduct operational and clinical research and to study temporal trends and the impact of changes in guidelines and other interventions.
- The development of a standardised Data Exchange Standard protocol has contributed to increase data quality, and data have been enriched by linkage to cancer registries, vital registries and administrative databases.
- Collaborations with the World Health Organization, UNAIDS, the mathematical modelling community and other consortia have ensured that the analyses of the African IeDEA regions contributed substantially to global health policy and decision making.
- Weaknesses include the limitations inherent in secondary use of routine clinical care data, with missing data, the lack of standardised follow-up visits, and substantial loss to follow-up resulting in unknown outcome.
- Additional challenges include the lack of population-based samples, including HIV negative controls or ART-naive comparison groups, and the lack of measurements such as non-communicable disease risk factors and outcomes. Dealing with risk factors that affect clinical decision making, and the need to control for (time-dependent) confounding can also be challenging.

For peer review only - http://bmjopen.bmj.com/site/about/guidelines.xhtml

REFERENCES

1 UNAIDS. Global AIDS update 2016. Geneva, Switzerland: 2016.

2 UNAIDS. UNAIDS Data 2018. UNAIDS https://www.aidsdatahub.org/unaids-data-2018-unaids-2018

3 UNAIDS. 90-90-90: An ambitious treatment target to help end the AIDS epidemic. Geneva, Switzerland: 2014.

4 UNAIDS. Ending AIDS. Progress towards the 90-90-90- targets. Geneva, Switzerland: 2017.

5 Egger M, Ekouevi DK, Williams C, *et al.* Cohort Profile: The international epidemiological databases to evaluate AIDS (IeDEA) in sub-Saharan Africa. *Int J Epidemiol* 2012;**41**:1256–64. doi:10.1093/ije/dyr080

6 Harris PA, Taylor R, Minor BL, *et al.* The REDCap consortium: Building an international community of software platform partners. *J Biomed Inform* 2019;**95**:103208. doi:10.1016/j.jbi.2019.103208

7 Duda SNSN, Farr AMAM, Lindegren MLML, *et al.* Characteristics and comprehensiveness of adult HIV care and treatment programmes in Asia-Pacific, sub-Saharan Africa and the Americas: results of a site assessment conducted by the International epidemiologic Databases to Evaluate AIDS Collaboration. *J Int AIDS Soc* 2014;**17**:19045. doi:10.7448/IAS.17.1.19045

8 Parcesepe AM, Mugglin C, Nalugoda F, *et al.* Screening and management of mental health and substance use disorders in HIV treatment settings in low- and middle-income countries within the global IeDEA consortium. *J Int AIDS Soc* 2018;**21**:e25101. doi:10.1002/jia2.25101

9 Ballif M, Nhandu V, Wood R, *et al.* Detection and management of drug-resistant tuberculosis in HIV-infected patients in lower-income countries. *Int J Tuberc Lung Dis Off J Int Union Tuberc Lung Dis* 2014;**18**:1327–36. doi:10.5588/ijtld.14.0106

10 Fenner L, Forster M, Boulle A, *et al.* Tuberculosis in HIV programmes in lower-income countries: practices and risk factors. *Int J Tuberc Lung Dis Off J Int Union Tuberc Lung Dis* 2011;**15**:620–7. doi:10.5588/ijtld.10.0249

11 Ballif M, Renner L, Claude Dusingize J, *et al.* Tuberculosis in Pediatric Antiretroviral Therapy Programs in Low- and Middle-Income Countries: Diagnosis and Screening Practices. *J Pediatr Infect Dis Soc* 2015;**4**:30–8. doi:10.1093/jpids/piu020

12 Horner M-J, Chasimpha S, Spoerri A, *et al.* High Cancer Burden Among Antiretroviral Therapy Users in Malawi: a Record Linkage Study of Observational HIV Cohorts and Cancer Registry Data. *Clin Infect Dis* Published Online First: 17 November 2018. doi:10.1093/cid/ciy960

13 Dhokotera T, Bohlius J, Spoerri A, *et al.* The burden of cancers associated with HIV in the South African public health sector, 2004-2014: a record linkage study. *Infect Agent Cancer* 2019;**14**:12. doi:10.1186/s13027-019-0228-7

For peer review only - http://bmjopen.bmj.com/site/about/guidelines.xhtml

14 Boulle A, Schomaker M, May MT, *et al.* Mortality in patients with HIV-1 infection starting antiretroviral therapy in South Africa, Europe, or North America: a collaborative analysis of prospective studies. *PLoS Med* 2014;**11**:e1001718. doi:10.1371/journal.pmed.1001718

15 Davies M-A, Tsondai P, Tiffin N, *et al.* Where do HIV-infected adolescents go after transfer? - Tracking transition/transfer of HIV-infected adolescents using linkage of cohort data to a health information system platform. *J Int AIDS Soc* 2017;**20**:21668. doi:10.7448/IAS.20.4.21668

16 WHO. Consolidated guidelines on the use of antiretroviral drugs for treating and preventing HIV infection: recommendations for a public health approach. Geneva, Switzerland: 2013.

17 (PEPFAR). PEPFAR 2018 Country Operational Plan Guidance for Standard Process Countries Table of Contents. 2018.

18 Cottrell ML, Hadzic T, Kashuba ADM. Clinical Pharmacokinetic, Pharmacodynamic and Drug-Interaction Profile of the Integrase Inhibitor Dolutegravir. *Clin Pharmacokinet* 2013;**52**:981–94. doi:10.1007/s40262-013-0093-2

19 Mesplede T, Quashie PK, Osman N, *et al.* Viral fitness cost prevents HIV-1 from evading dolutegravir drug pressure. *Retrovirology* 2013;**10**:22. doi:10.1186/1742-4690-10-22

20 Zash R, Holmes L, Diseko M, *et al.* Neural-Tube Defects and Antiretroviral Treatment Regimens in Botswana. *N Engl J Med* 2019;:NEJMoa1905230. doi:10.1056/NEJMoa1905230

21 Zaniewski E, Tymejczyk O, Kariminia A, *et al.* IeDEA-WHO Research-Policy Collaboration: contributing real-world evidence to HIV progress reporting and guideline development. *J Virus Erad* 2018;**4**:9–15.

22 Anderegg N, Johnson LF, Zaniewski E, *et al.* All-cause mortality in HIV-positive adults starting combination antiretroviral therapy: correcting for loss to follow-up. *AIDS Lond Engl* 2017;**31 Suppl 1**:S31–40. doi:10.1097/QAD.0000000000001321

23 Mahy M, Penazzato M, Ciaranello A, *et al.* Improving estimates of children living with HIV from the Spectrum AIDS Impact Model. *AIDS Lond Engl* 2017;**31 Suppl 1**:S13–22. doi:10.1097/QAD.0000000000001306

24 Davies M-AM-AA, May M, Bolton-Moore C, *et al.* Prognosis of Children With HIV-1 Infection Starting Antiretroviral Therapy in Southern Africa. *Pediatr Infect Dis J* 2014;**33**:608–16. doi:10.1097/INF.0000000000000214

25 Petersen ML, Tran L, Geng EH, *et al.* Delayed switch of antiretroviral therapy after virologic failure associated with elevated mortality among HIV-infected adults in Africa. *AIDS Lond Engl* 2014;**28**:2097–107. doi:10.1097/QAD.0000000000000349

26 Tchounga BK, Hønge BL, Eholie SP, *et al.* Effect of sex and age on outcomes among HIV-2-infected patients starting antiretroviral therapy in West Africa. *AIDS Lond Engl* 2016;**30**:2707–14. doi:10.1097/QAD.0000000000001232

27 Giles ML, Achhra AC, Abraham AG, *et al.* Sex-based differences in antiretroviral therapy initiation, switching and treatment interruptions: global overview from the International

For peer review only - http://bmjopen.bmj.com/site/about/guidelines.xhtml

Epidemiologic Databases to Evaluate AIDS (IeDEA). *J Int AIDS Soc* 2018;**21**:e25149. doi:10.1002/jia2.25149

28 Haas AD, Keiser O, Balestre E, *et al.* Monitoring and switching of first-line antiretroviral therapy in adult treatment cohorts in sub-Saharan Africa: Collaborative analysis. *Lancet HIV* 2015;**2**:e271–e278. doi:10.1016/S2352-3018(15)00087-9

29 Chimbetete C, Katzenstein D, Shamu T, *et al.* HIV-1 Drug Resistance and Third-Line Therapy Outcomes in Patients Failing Second-Line Therapy in Zimbabwe. *Open Forum Infect Dis* 2018;**5**:1–8. doi:10.1093/ofid/ofy005

30 Wools-Kaloustian K, Marete I, Ayaya S, *et al.* Time to First-Line ART Failure and Time to Second-Line ART Switch in the IeDEA Pediatric Cohort. *Jaids-J Acquir Immune Defic Syndr* 2018;**78**:221–30. doi:10.1097/QAI.0000000000001667

31 Charpentier C, Eholié S, Anglaret X, *et al.* Genotypic resistance profiles of HIV-2-treated patients in West Africa. *AIDS Lond Engl* 2014;**28**:1161–9. doi:10.1097/QAD.0000000000000244

32 Kiragga AN, Lok JJ, Musick BS, *et al.* CD4 trajectory adjusting for dropout among HIV-positive patients receiving combination antiretroviral therapy in an East African HIV care centre. *J Int AIDS Soc* 2014;**17**:18957. doi:10.7448/IAS.17.1.18957

33 Geng EH, Neilands TB, Thièbaut R, *et al.* CD41 T cell recovery during suppression of HIV replication: an international comparison of the immunological efficacy of antiretroviral therapy in North America, Asia and Africa. *Int J Epidemiol* 2015;**44**:251–63. doi:10.1093/ije/dyu271

34 Technau K-G, Schomaker M, Kuhn L, *et al.* Virologic response in children treated with abacavir-compared with stavudine-based antiretroviral treatment: a South African multi-cohort analysis. *Pediatr Infect Dis J* 2014;**33**:617–22. doi:10.1097/INF.0000000000000222

35 Tenthani L, Haas AD, Tweya H, *et al.* Retention in care under universal antiretroviral therapy for HIV-infected pregnant and breastfeeding women (‘Option B+’) in Malawi. *AIDS* 2014;**28**:589–598 Keywords:

36 Collaborative Initiative for Paediatric HIV Education and Research (CIPHER) Global Cohort Collaboration, Slogrove AL, Schomaker M, *et al.* The epidemiology of adolescents living with perinatally acquired HIV: A cross-region global cohort analysis. *PLoS Med* 2018;**15**:e1002514. doi:10.1371/journal.pmed.1002514

37 Kariminia A, Law M, Davies M, *et al.* Mortality and losses to follow-up among adolescents living with HIV in the Ie DEA global cohort collaboration. *J Int AIDS Soc* 2018;**21**:e25215. doi:10.1002/jia2.25215

38 Rosen S, Fox MP, Gill CJ. Patient retention in antiretroviral therapy programs in sub-Saharan Africa: a systematic review. *PLoSMed* 2007;**4**:e298.

39 Brinkhof MWGWMWG, Pujades-Rodriguez M, Egger M. Mortality of patients lost to follow-up in antiretroviral treatment programmes in resource-limited settings: systematic review and meta-analysis. *PLoS One* 2009;**4**:e5790. doi:10.1371/journal.pone.0005790

For peer review only - http://bmjopen.bmj.com/site/about/guidelines.xhtml

40 Geng EH, Odeny TA, Lyamuya RE, *et al.* Estimation of mortality among HIV-infected people on antiretroviral treatment in east Africa: a sampling based approach in an observational, multisite, cohort study. *Lancet HIV* 2015;**2**:e107–16. doi:10.1016/S2352-3018(15)00002-8

41 An M-W, Frangakis CE, Yiannoutsos CT. Choosing profile double-sampling designs for survival estimation with application to President's Emergency Plan for AIDS Relief evaluation. *Stat Med* 2014;**33**:2017–29. doi:10.1002/sim.6087

42 Schomaker M, Gsponer T, Estill J, *et al.* Non-ignorable loss to follow-up: correcting mortality estimates based on additional outcome ascertainment. *Stat Med* 2014;**33**:129–142. doi:10.1002/sim.5912

43 Brinkhof MWG, Spycher BD, Yiannoutsos C, *et al.* Adjusting mortality for loss to follow-up: Analysis of five art programmes in sub-saharan africa. *PLoS ONE* 2010;**5**:3–8. doi:10.1371/journal.pone.0014149

44 Egger M, Spycher BD, Sidle J, *et al.* Correcting mortality for loss to follow-up: a nomogram applied to antiretroviral treatment programmes in sub-Saharan Africa. *PLoS Med* 2011;**8**:e1000390. doi:10.1371/journal.pmed.1000390

45 Yiannoutsos CT, Johnson LF, Boulle A, *et al.* Estimated mortality of adult HIV-infected patients starting treatment with combination antiretroviral therapy. *Sex Transm Infect* 2012;**88 Suppl 2**:i33—-43.

46 Haas AD, Zaniewski E, Anderegg N, *et al.* Retention and mortality on antiretroviral therapy in sub-Saharan Africa: collaborative analyses of HIV treatment programmes. *J Int AIDS Soc* 2018;**21**:e25084. doi:10.1002/jia2.25084

47 Hector J, Vinikoor M, Chilengi R, *et al.* No Impact of Hepatitis B Virus Infection on Early Mortality Among Human Immunodeficiency Virus–Infected Patients in Southern Africa. *Clin Infect Dis* 2018;**67**:1310–1311. doi:10.1093/cid/ciy304

48 Wandeler G, Mulenga L, Hobbins M, *et al.* Absence of Active Hepatitis C Virus Infection in Human Immunodeficiency Virus Clinics in Zambia and Mozambique. *Open Forum Infect Dis* 2016;**3**:ofw049. doi:10.1093/ofid/ofw049

49 Jaquet A, Wandeler G, Nouaman M, *et al.* Alcohol use, viral hepatitis and liver fibrosis among HIV-positive persons in West Africa: A cross-sectional study. *J Int AIDS Soc* 2017;**20**:21424. doi:10.7448/IAS.20.1.21424

50 Rohner E, Bütikofer L, Schmidlin K, *et al.* Cervical cancer risk in women living with HIV across four continents: A multicohort study: Regional cervical cancer risk in HIV-positive women. *Int J Cancer* Published Online First: 19 June 2019. doi:10.1002/ijc.32260

51 Brown SA, Abbas S, Davies M-A, *et al.* Brief Report: Pediatric Cancer Burden and Treatment Resources Within the Pediatric IeDEA Consortium. *J Acquir Immune Defic Syndr 1999* 2017;**76**:60–4. doi:10.1097/QAI.0000000000001453

52 Semeere A, Wenger M, Busakhala N, *et al.* A prospective ascertainment of cancer incidence in sub-Saharan Africa: The case of Kaposi sarcoma. *Cancer Med* 2016;**5**:914–28. doi:10.1002/cam4.618

For peer review only - http://bmjopen.bmj.com/site/about/guidelines.xhtml

53 Carlucci JG, Blevins Peratikos M, Kipp AM, *et al.* Tuberculosis Treatment Outcomes Among HIV/TB-Coinfected Children in the International Epidemiology Databases to Evaluate AIDS (IeDEA) Network. *J Acquir Immune Defic Syndr 1999* 2017;**75**:156–63. doi:10.1097/QAI.0000000000001335

54 Zurcher K, Ballif M, Fenner L, *et al.* Drug susceptibility testing and mortality in patients treated for tuberculosis in high-burden countries: a multicentre cohort study. *Lancet Infect Dis* 2019;**19**:298–307. doi:10.1016/S1473-3099(18)30673-X

55 Gygli SM, Keller PM, Ballif M, *et al.* Whole-Genome Sequencing for Drug Resistance Profile Prediction in Mycobacterium tuberculosis. *Antimicrob Agents Chemother* 2019;**63**:e02175-18. doi:10.1128/AAC.02175-18

56 Zürcher K, Ballif M, Kiertiburanakul S, *et al.* Diagnosis and clinical outcomes of extrapulmonary tuberculosis in antiretroviral therapy programmes in low- and middle-income countries: a multicohort study. *J Int AIDS Soc* 2019;**22**:e25392. doi:10.1002/jia2.25392

57 Mulenga L, Musonda P, Mwango A, *et al.* Effect of baseline renal function on tenofovir-containing antiretroviral therapy outcomes in Zambia. *Clin Infect Dis Off Publ Infect Dis Soc Am* 2014;**58**:1473–80. doi:10.1093/cid/ciu117

58 Rohner E, Schmidlin K, Zwahlen M, *et al.* Kaposi Sarcoma Risk in HIV-Infected Children and Adolescents on Combination Antiretroviral Therapy From Sub-Saharan Africa, Europe, and Asia. *Clin Infect Dis* 2016;**63**:ciw519. doi:10.1093/cid/ciw519

59 Chêne G, Phillips A, Costagliola D, *et al.* Cohort profile: Collaboration of Observational HIV Epidemiological Research Europe (COHERE) in EuroCoord. *Int J Epidemiol* 2017;**46**:797. doi:10.1093/ije/dyw211

60 Tymejczyk O, Brazier E, Yiannoutsos CT, *et al.* Changes in rapid HIV treatment initiation after national “treat all” policy adoption in 6 sub-Saharan African countries: Regression discontinuity analysis. *PLOS Med* 2019;**16**:e1002822. doi:10.1371/journal.pmed.1002822

61 Yotebieng M, Brazier E, Addison D, *et al.* Research priorities to inform “Treat All” policy implementation for people living with HIV in sub-Saharan Africa: a consensus statement from the International epidemiology Databases to Evaluate AIDS (Ie DEA). *J Int AIDS Soc* 2019;**22**:e25218. doi:10.1002/jia2.25218

62 Nash D, Yotebieng M, Sohn AH. Treating all people living with HIV in sub-Saharan Africa: a new era calling for new approaches. *J Virus Erad* 2018;**4**:1–4.

63 Enane LA, Davies M-A, Leroy V, *et al.* Traversing the cascade: urgent research priorities for implementing the “treat all” strategy for children and adolescents living with HIV in sub-Saharan Africa. *J Virus Erad* 2018;**4**:40–46.

64 Abuogi LL, Humphrey JM, Mpody C, *et al.* Achieving UNAIDS 90-90-90 targets for pregnant and postpartum women in sub-Saharan Africa: progress, gaps and research needs. *J Virus Erad* 2018;**4**:33–9.

65 Lancaster KE, Hetrick A, Jaquet A, *et al.* Substance use and universal access to HIV testing and treatment in sub-Saharan Africa: implications and research priorities. *J Virus Erad* 2018;**4**:26–32.

For peer review only - http://bmjopen.bmj.com/site/about/guidelines.xhtml

66 de Waal R, Lessells R, Hauser A, *et al.* HIV drug resistance in sub-Saharan Africa: public health questions and the potential role of real-world data and mathematical modelling. *J Virus Erad* 2018;**4**:55–8.

67 Panayidou K, Davies M-A, Anderegg N, *et al.* Global temporal changes in the proportion of children with advanced disease at the start of combination antiretroviral therapy in an era of changing criteria for treatment initiation. *J Int AIDS Soc* 2018;**21**:e25200. doi:10.1002/jia2.25200

68 Anderegg N, Panayidou K, Abo Y, *et al.* Global Trends in CD4 Cell Count at the Start of Antiretroviral Therapy: Collaborative Study of Treatment Programs. *Clin Infect Dis* 2018;**66**:893–903. doi:10.1093/cid/cix915

69 Collaborative Initiative for Paediatric HIV Education and Research (CIPHER) Global Cohort Collaboration. Incidence of switching to second-line antiretroviral therapy and associated factors in children with HIV: an international cohort collaboration. *Lancet HIV* 2019;**6**:e105–15. doi:10.1016/S2352-3018(18)30319-9

For peer review only

For peer review only - http://bmjopen.bmj.com/site/about/guidelines.xhtml

Table 1: Characteristics of 240 facilities providing ART in the African regions if the IeDEA (source: site assessment survey 2017).

	West Africa	Central Africa	East Africa	Southern Africa	All regions (%)
**No of active facilities**	17	19	72	132	240
**Location**					
Urban	17	19	19	65	120 (50)
Rural	0	0	52	67	119 (50)
**Level of care**					
Primary	4	12	49	107	172 (74)
Secondary	1	0	16	20	37 (16)
Tertiary	12	7	6	5	24 (10)
**Type of facility**					
Public	12	16	69	128	225 (95)
Private	2	3	3	4	12 (5)
**Viral load**					
Routine testing	12	16	46	74	148 (62)
Results available within 15 days	6	13	11	22	52 (22)
Results available within 16-30 days	7	4	11	104	126 (53)
Tests performed onsite	8	6	4	3	21 (9)
Tests performed offsite	6	13	53	127	199 (83)
**CD4 monitoring**					
Routine testing	14	6	9	97	126 (53)
Results available same day	10	8	21	17	56 (23)
Tests performed onsite	11	8	23	51	93 (39)
Tests performed offsite	3	11	35	71	120 (50)
**HIV-1 genotypic drug resistance**					
Routine testing	2	2	10	48	62 (26)
**Routine tracing of LTFU**					
Yes	13	19	56	93	181 (75)
No	1	0	1	37	39 (16)
**Tracing method***					
Phone	14	18	57	110	199
SMS/mail/email	2	2	10	14	28
Home visit	9	17	52	129	207
**Medication disruption/stock outs last 12 m**					
Pharmacy available on site	14	19	56	96	185
First-line ART	5	2	20	32	59
Second-line ART	5	6	18	16	45

*Sites may use more than 1 method

For peer review only - http://bmjopen.bmj.com/site/about/guidelines.xhtml

For peer review only

Table 2: Proportion of patients with different nucleoside and non-nucleoside reverse transcriptase inhibitors regimen at ART start over time, by region.

	Central Africa								Eastern Africa							
	2001-2002	2003-2004	2005-2006	2007-2008	2009-2010	2011-2012	2013-2014	2015-2016	2001-2002	2003-2004	2005-2006	2007-2008	2009-2010	2011-2012	2013-2014	2015-2016
**NRTI**																
FTC+TDF	-	1.2	4.4	0.8	3.5	5.0	6.6	9.2	0.8	5.4	0.3	1.0	0.9	1.1	1.4	0.7
3TC+TDF	-	6.4	4.1	3.9	34.4	49.7	60.1	76.5	2.7	5.0	1.1	2.3	9.3	55.1	77.5	89.7
3TC+D4T	-	60.0	55.6	33.7	8.8	2.7	0.1	0.0	78.9	80.0	83.2	54.1	37.4	3.0	0.5	0.1
3TC+AZT	-	29.6	32.7	54.9	48.6	35.9	27.0	6.6	14.0	9.0	14.8	41.8	46.4	35.3	16.6	6.4
3TC+ABC	-	1.1	1.4	2.1	2.8	4.1	5.1	7.3	0.6	0.3	0.4	0.8	5.9	5.4	4.0	3.2
Other	-	1.8	1.8	4.7	1.9	2.6	1.1	0.4	3.1	0.4	0.2	0.1	0.2	0.1	0.0	0.0
**NNRTI**																
NVP	-	64.1	65	73.6	66.6	49.2	22.5	6.3	73.1	82.6	80.9	76.4	72.2	50.4	21.3	8.1
EFV	-	35.7	34.3	25.8	32.9	50.1	76.9	93.4	24.8	15.4	17.7	22.6	27.2	49.3	78.4	91.5
Other	-	0.2	0.7	0.7	0.5	0.7	0.6	0.2	2.0	2.0	1.4	1.0	0.6	0.3	0.4	0.4

	Southern Africa								West Africa							
	2001-2002	2003-2004	2005-2006	2007-2008	2009-2010	2011-2012	2013-2014	2015-2016	2001-2002	2003-2004	2005-2006	2007-2008	2009-2010	2011-2012	2013-2014	2015-2016
**NRTI**																
FTC+TDF	2.0	0.5	0.5	17.4	24.9	34.4	62.2	51.9	0.6	0.0	0.1	1.5	11.5	17.6	11.5	11.6
3TC+TDF	2.5	1.1	1.1	2.1	17.1	33.5	22.2	40.3	0.0	0.0	0.2	0.6	3.5	10.3	32.1	55.3
3TC+D4T	37.2	47.7	55.3	49.5	36.5	15.3	3.4	0.2	39.2	46.8	51.6	42.6	21.6	4.8	0.6	0.0
3TC+AZT	47.0	39.7	27.7	16.2	10.7	8.7	6.8	2.5	35.7	39.7	43.3	51.0	46.2	53.4	49.3	22.5
3TC+ABC	0.7	0.2	0.3	1.2	5.0	6.0	4.9	5.0	0.2	0.1	0.4	0.9	0.9	4.1	5.8	10.3
Other	10.6	10.9	15.1	13.6	5.8	2.2	0.5	0.1	24.3	13.4	4.5	3.4	16.3	9.8	0.6	0.3
**NNRTI**																
NVP	51.1	58.1	64.8	55.4	47.2	36.7	15.6	3.7	10.1	12.0	40.9	46.1	50.2	47.0	36.1	15.0
EFV	43.7	37.8	31.4	41.5	50.0	61.2	82.4	93.9	50.1	60.6	46.7	44.4	39.7	41.5	53.8	70.7
Other	5.2	4.1	3.7	3.1	2.9	2.1	1.9	2.4	39.8	27.4	12.5	9.6	10.2	11.5	10.1	14.3

Abbreviation: NRTI, nucleoside reverse transcriptase inhibitor; NNRTI, non-nucleoside reverse transcriptase inhibitor

For peer review only

For peer review only - http://bmjopen.bmj.com/site/about/guidelines.xhtml

Legends to Figures

Figure 1: Map of the 240 active facilities participating in the four African regions of the International epidemiology Databases to Evaluate AIDS (A), together with cumulative numbers of patients starting antiretroviral therapy (B).

Figure 2: Daily number of CD4 cell counts and viral load measurements over time (bar chart) and the number of patients in care (red line).

Figure 3. Trends in mortality (A) and loss to follow (B), 2001 to 2016.

For peer review only

For peer review only - http://bmjopen.bmj.com/site/about/guidelines.xhtml

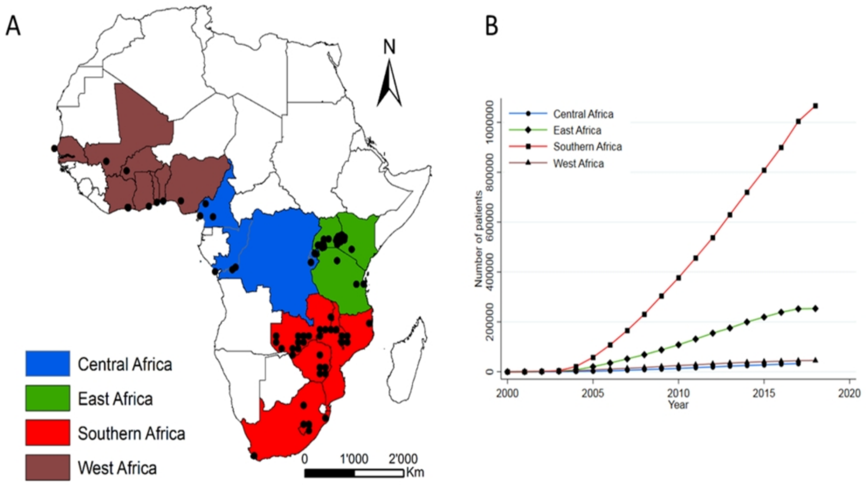

Figure 1. Map of the 240 active facilities participating in the four African regions of the International epidemiology Databases to Evaluate AIDS (A), together with cumulative numbers of patients starting antiretroviral therapy (B).

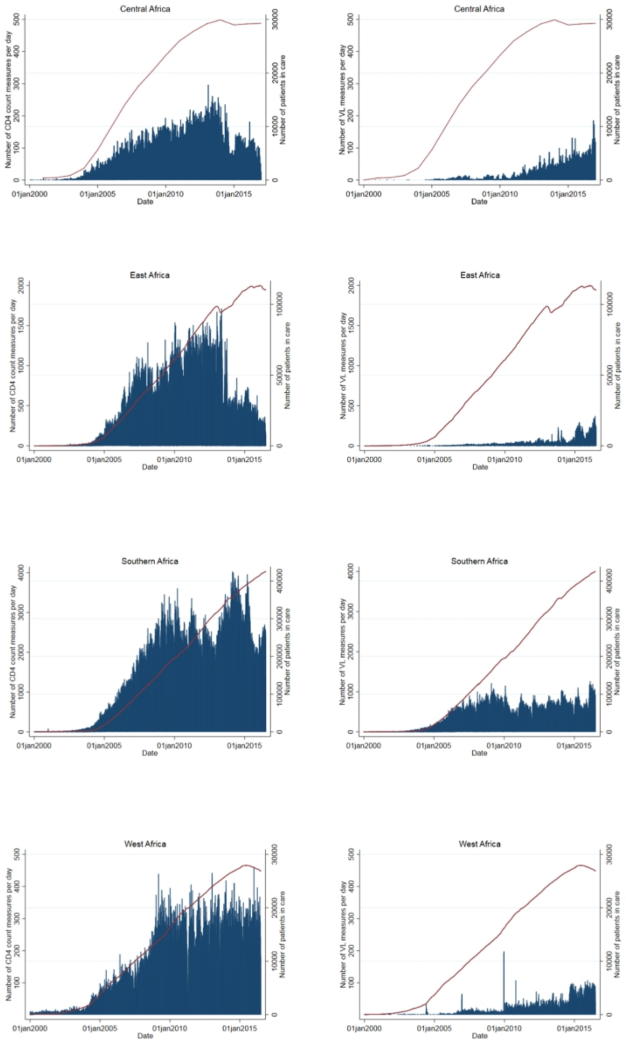

Figure 2. Daily number of CD4 cell counts and viral load measurements over time (bar chart) and the number of patients in care (red line).

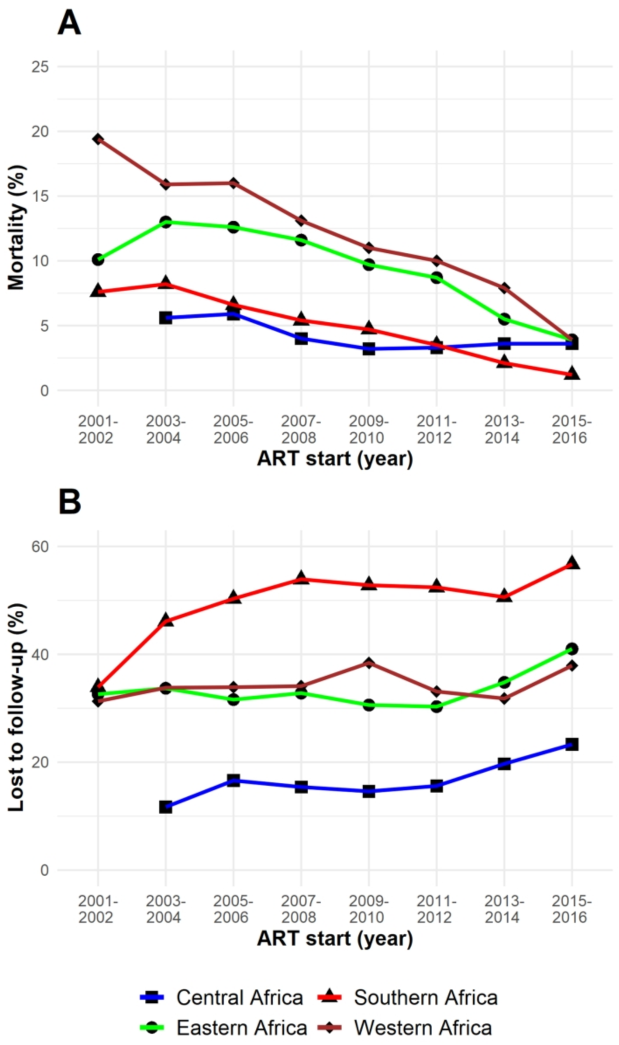

Figure 3. Trends in mortality (A) and loss to follow (B), 2001 to 2016.

BMJ Open

Cohort profile: The International epidemiology Databases to Evaluate AIDS (IeDEA) in sub-Saharan Africa, 2012-2019

Journal:	*BMJ Open*
Manuscript ID	bmjopen-2019-035246.R1
Article Type:	Cohort profile
Date Submitted by the Author:	21-Feb-2020
Complete List of Authors:	Chammartin, Frédérique; University of Bern, Institute of Social & Preventive Medicine<br>Dao Ostinelli, Cam Ha; University of Bern, Institute of Social & Preventive Medicine<br>Anastos, Kathryn; Yeshiva University Albert Einstein College of Medicine, Department of Medicine<br>Jaquet, Antoine; University of Bordeaux, ISPED, Centre INSERM U1219 – Bordeaux Population Health<br>Brazier, Ellen ; City University of New York, Institute for Implementation Science in Population Health; City University of New York, Graduate School of Public Health and Health Policy<br>Brown, Steven; Indiana University Richard M Fairbanks School of Public Health, Department of Biostatistics<br>DABIS, FRANCOIS; University of Bordeaux, ISPED, Centre INSERM U1219 – Bordeaux Population Health<br>Davies, Mary-Ann; University of Cape Town, Centre for Infectious Disease Epidemiology and Research, School of Public Health and Family Medicine<br>Duda, Stephany N; Vanderbilt University School of Medicine, Department of Biomedical Informatics<br>Malateste, Karen; University of Bordeaux, ISPED, Centre INSERM U1219 – Bordeaux Population Health<br>Nash, Denis; City University of New York, Institute for Implementation Science in Population Health; City University of New York, Graduate School of Public Health and Health Policy<br>Wools-Kaloustian, Kara; Indiana University System, Department of Medicine<br>von Groote, Per M; University of Bern, Institute of Social & Preventive Medicine<br>Egger, Matthias; University of Bern, Institute of Social & Preventive Medicine
**Primary Subject Heading**:	HIV/AIDS
Secondary Subject Heading:	Epidemiology, Global health, Health services research, Infectious diseases
Keywords:	HIV & AIDS < INFECTIOUS DISEASES, EPIDEMIOLOGY, INFECTIOUS DISEASES, Tuberculosis < INFECTIOUS DISEASES

I, the Submitting Author has the right to grant and does grant on behalf of all authors of the Work (as defined in the below author licence), an exclusive licence and/or a non-exclusive licence for contributions from authors who are: i) UK Crown employees; ii) where BMJ has agreed a CC-BY licence shall apply, and/or iii) in accordance with the terms applicable for US Federal Government officers or employees acting as part of their official duties; on a worldwide, perpetual, irrevocable, royalty-free basis to BMJ Publishing Group Ltd ("BMJ") its licensees and where the relevant Journal is co-owned by BMJ to the co-owners of the Journal, to publish the Work in this journal and any other BMJ products and to exploit all rights, as set out in our licence.

The Submitting Author accepts and understands that any supply made under these terms is made by BMJ to the Submitting Author unless you are acting as an employee on behalf of your employer or a postgraduate student of an affiliated institution which is paying any applicable article publishing charge ("APC") for Open Access articles. Where the Submitting Author wishes to make the Work available on an Open Access basis (and intends to pay the relevant APC), the terms of reuse of such Open Access shall be governed by a Creative Commons licence – details of these licences and which Creative Commons licence will apply to this Work are set out in our licence referred to above.

Other than as permitted in any relevant BMJ Author's Self Archiving Policies, I confirm this Work has not been accepted for publication elsewhere, is not being considered for publication elsewhere and does not duplicate material already published. I confirm all authors consent to publication of this Work and authorise the granting of this licence.

For peer review only

Revision 1 for BMJ Open

Cohort profile: The International epidemiology Databases to Evaluate AIDS (IeDEA) in sub-Saharan Africa, 2012-2019

Frédérique Chammartin[1], Cam Ha Dao Ostinelli[1], Kathryn Anastos[2], Antoine Jaquet,[3] Ellen Brazier[4,5], Steven Brown[6], François Dabis[3], Mary-Ann Davies[7], Stephany N Duda[8], Karen Malateste[3], Denis Nash[4,5], Kara K Wools-Kaloustian[9], Per M von Groote[1], Matthias Egger[1,7]

[1] Institute of Social and Preventive Medicine, University of Bern, Bern, Switzerland
[2] Department of Medicine, Albert Einstein College of Medicine, Bronx, NY, USA
[3] Université Bordeaux, ISPED, Centre INSERM U1219 – Bordeaux Population Health, Bordeaux, France
[4] Institute for Implementation Science in Population Health, City University of New York, NY, USA
[5] Graduate School of Public Health and Health Policy, City University of New York, NY, USA
[6] Department of Biostatistics, Indiana University Fairbanks School of Public Health, Indianapolis, IN, United States
[7] Centre for Infectious Disease Epidemiology and Research, School of Public Health and Family Medicine, University of Cape Town, South Africa
[8] Department of Biomedical Informatics, Vanderbilt School of Medicine, Nashville, TN, USA
[9] Department of Medicine, Indiana University School of Medicine, Indianapolis, IN, United States

Correspondence to:

Professor Matthias Egger
Institute of Social and Preventive Medicine (ISPM)
University of Bern
Mittelstrasse 43
CH-3012 Bern
Switzerland
matthias.egger@ispm.unibe.ch

Abstract 293 words, main text 2426 words, 2 tables, 3 figures, 1 Box with strengths and weaknesses (176 words), further details (424 words), 69 references

For peer review only - http://bmjopen.bmj.com/site/about/guidelines.xhtml

ABSTRACT

Purpose: The objectives of the International epidemiology Databases to Evaluate AIDS (IeDEA) are to (i) evaluate the delivery of combination antiretroviral therapy (ART) in children, adolescents and adults in sub-Saharan Africa, (ii) to describe ART regimen effectiveness, durability and tolerability, (iii) to examine HIV-related comorbidities and co-infections, and (iv) to examine the pregnancy- and HIV-related outcomes of women on ART and their infants exposed to HIV or antiretroviral therapy in utero or via breastmilk.

Participants: IeDEA is organized in four regions (Central, East, Southern and West Africa), with 240 treatment and care sites, six data centres at African, European and US universities, and almost 1.4 million children, adolescents and adult people living with HIV (PLWHIV) enrolled.

Findings to date: The data include socio-demographic characteristics, clinical outcomes, opportunistic events, treatment regimens, clinic visits and laboratory measurements. They have been used to analyse outcomes in people living with HIV-1 or HIV-2 who initiate ART, including determinants of mortality, of switching to second-line and third-line ART, drug resistance, loss to follow-up and the immunological and virological response to different ART regimens. Programme-level estimates of mortality have been corrected for loss to follow-up. We examined the impact of co-infection with hepatitis B and C, and the epidemiology of different cancers and of (multi-drug resistant) tuberculosis, renal disease and of mental illness. The adoption of "Treat All", making ART available to all PLWHIV regardless of $CD4^+$ cell count or clinical stage was another important research topic.

Future plans: IeDEA has formulated several research priorities for the "Treat All" era in sub-Saharan Africa. It recently obtained funding to set up sentinel sites where additional data are prospectively collected on cardiometabolic risks factors as well as mental health and liver diseases, and is planning to create a drug resistance database.

For peer review only - http://bmjopen.bmj.com/site/about/guidelines.xhtml

For peer review only

Strengths and limitations of this study

- An important strength of the IeDEA cohort collaboration in sub-Saharan Africa is its large size, which allows analyses of outcomes of antiretroviral therapy (ART) in children, adolescents and pregnant and postpartum women, across diverse settings.
- The data reflect routine care across a wide range of real-world settings during the scale up of ART in sub-Saharan Africa and thus provide a valuable platform to conduct operational and clinical research and to study temporal trends and the impact of changes in guidelines and other interventions.
- The development of a standardised Data Exchange Standard protocol has contributed to increase data quality, and data have been enriched by linkage to cancer registries, vital registries and administrative databases.
- Collaborations with the World Health Organization, UNAIDS, the mathematical modelling community and other consortia have ensured that the analyses of the African IeDEA regions contributed to global health policy and decision making.
- Weaknesses include the limitations inherent in secondary use of routine clinical care data, with missing data, the lack of standardised follow-up visits, and substantial loss to follow-up resulting in unknown outcome.

For peer review only

For peer review only - http://bmjopen.bmj.com/site/about/guidelines.xhtml

INTRODUCTION

The roll-out of combination antiretroviral therapy (ART) in sub-Saharan Africa from 2004 onwards has substantially improved the prognosis of HIV-1 infection, with a decline in AIDS-related deaths [1] and a decline in the incidence of new HIV-1 infections [2]. However, in many settings HIV/AIDS is still a public health threat. An estimated 1.8 million new infections occurred in 2017 and almost a million adult and child deaths were due to HIV, most of them in sub-Saharan Africa [2].

The World Health Organization (WHO), the Joint United Nations Programme on HIV/AIDS (UNAIDS) and many of the countries most heavily affected by the HIV epidemic have committed to ending HIV/AIDS as a major public health problem by 2030.[1] Targets to be reached by 2020 include that 90% of people living with HIV (PLWHIV) be aware of their status, 90% of those diagnosed initiate ART, and 90% of those on ART achieve undetectable viral loads (the 90-90-90 targets) [3]. Progress towards these goals has been more substantial in Eastern and Southern Africa than in West and Central Africa. Of the 20.6 million PLWHIV in Eastern and Southern Africa, an estimated 58% were virally suppressed, compared to 39% of 5.0 million in West and Central Africa [4].

Established more than ten years ago by the National Institutes of Health (NIH), the International epidemiology Databases to Evaluate AIDS (IeDEA) are a global cohort collaboration that collects HIV/AIDS data from HIV care and treatment programs, including in sub-Saharan Africa. The regional IeDEA data centres consolidate, curate and analyse data to evaluate the outcomes of people living with HIV/AIDS and monitor progress. The first years of the cohorts in sub-Saharan Africa were described previously [5]; here we provide an update on methods, key data and future plans.

COHORT DESCRIPTION

In 2006 the National Institute of Allergy and Infectious Diseases (NIAID) sought applications for a global consortium structured through regional centres to pool clinical and epidemiological data on PLWHIV, in order to address questions that could not be answered by individual cohorts [5]. IeDEA covers seven geographic regions, namely North America, the Caribbean and Central/South America, the Asia-Pacific and four regions in sub-Saharan Africa: West Africa, Central Africa, East Africa and Southern

For peer review only

Africa. The project was initially funded for a 5-year period and has since been extended twice, with the current funding cycle ending in 2021.

Settings and number of PLWHIV enrolled

To date, the African regions of IeDEA received data from 240 HIV care and treatment facilities in 19 sub-Saharan African countries (Figure 1A). Close to 1,400,000 PLWHIV who initiated ART in sub-Saharan Africa are included (Figure 1B), of whom over 680,000 are currently in care. In East and Southern Africa, both urban and rural facilities are well represented, while in Central and West Africa, urban facilities dominate. Facilities are predominately public (94%) and operated at the primary or secondary care level, with the exception of West Africa where 70% of facilities are at the tertiary level of care (Table 1).

Data collection at individual and site level

Since its inception, IeDEA has collected routine clinical data of PLWHIV followed under treatment, which includes socio-demographic characteristics, clinical outcomes, opportunistic events, treatment regimens, clinic visits and laboratory measurements. More recently, the IeDEA network has developed a Data Exchange Standard (DES) protocol (see www.iedeades.org) with 25 data tables, which include a total of 228 unique variables (36 compulsory and 192 additional variables) and within-region unique patient research identifiers. Standardized data collection is supported by eight codebooks, including the Anatomical Therapeutic Chemical (ATC) classification for drugs and lists with codes for reasons for stopping treatment, for dropping out of the cohort, for mode of HIV infection, country, type and site of comorbidities, laboratory measurements and units of measurements and type of viral load assay. The collection, management and sharing of data is facilitated by the Harmonist toolkit, a software and standards package that supports research projects through the Research Electronic Data Capture (REDCap) system [6].

In recent years, site assessments and site surveys have been conducted on a regular basis to collect up-to-date information related to available clinical service and care models in the participating facilities. For example, a study compared the characteristics and comprehensiveness of adult HIV care and treatment programmes in sub-Saharan Africa with programmes in the Americas and Asia-Pacific region [7]. Other studies examined the

For peer review only - http://bmjopen.bmj.com/site/about/guidelines.xhtml

management of mental health and substance use disorders [8], or the diagnostic and screening practices for (drug resistant) tuberculosis in adult and paediatric patients [9–11]. Furthermore, the routine data collected by participating sites have been enriched in some countries by linking the HIV databases to cancer registries [12,13], vital registries [14] or administrative databases [15].

Trends in CD4 cell count and viral load measurements

While WHO continues to recommend a CD4 cell count before starting ART to inform the management of advanced disease and differentiated care in the Treat All era, it also recommends that CD4 testing be replaced by viral load measurement for monitoring of treatment and identification of treatment failure [16]. Figure 2 shows that in Southern and West Africa, the number of CD4 measurements tended to be stable over time, despite an increasing number of PLWHIV in care, while in East and Central Africa the number of CD4 measurements dropped. At present, 53% of the active facilities reported routine CD4 testing and 62% routine viral load testing (Table 1). The United States President's Emergency Plan for AIDS Relief (PEPFAR), which provides substantial funding for AIDS treatment, care and prevention in countries most affected by the epidemic, has progressively reduced its support for CD4 testing [17].

Trends in antiretroviral therapy

Until recently, the recommended first-line ART regimen in sub-Saharan Africa consisted of two nucleoside reverse transcriptase inhibitors and one non-nucleoside reverse transcriptase inhibitor (2NRTIs+1NNRTI). The combination of tenofovir (TDF), lamivudine (3TC) (or emtricitabine (FTC)) and efavirenz (EFV) is the current treatment of choice. The phasing out of stavudine (D4T) and nevirapine (NVP) was almost complete in 2014 (Table 2). East Africa and Southern Africa are currently rolling out dolutegravir (DTG), an integrase inhibitor with a high barrier to resistance [18][19]. Due to concerns about an increased risk of neural tube defects if taken during pregnancy [20], the roll-out to women has been delayed or limited in some settings. Of note, drug stock-outs in the last 12 months were reported by 59 facilities for first-line drugs, and by 45 for second-line drugs (Table 1).

For peer review only - http://bmjopen.bmj.com/site/about/guidelines.xhtml

Mortality and retention in care

In cohorts of PLWHIV who initiated ART in consecutive two-year periods from 2001 to 2016, mortality at 3 years declined substantially in all African IeDEA regions (Figure 3A). Loss to follow-up, defined as more than 90 days late to the next scheduled visit, remained substantial in all regions, and particularly high in Southern Africa (Figure 3B). Retention in care is key to the success of the public health approach to ART. Loss to follow-up has been an important issue for IeDEA, and activities to trace PLWHIV not returning to the clinic have increased in recent years. At present, tracing of PLWHIV on ART who were lost to follow-up is in place in 89% of the active facilities; 75% have implemented it routinely (Table 1). Tracing methods vary widely across facilities and include phone calls and home visits by clinic staff or community health workers.

Patient and public involvement

IeDEA is based on the collection of routine clinical data and no patients were involved in developing the research question, outcome measures and overall design of the collaboration. Due to the anonymous nature of the data, we cannot disseminate the results of analyses of the data directly to study participants.

FINDINGS TO DATE

Over 500 publications in MEDLINE acknowledge funding from a core grant from the NIH to one or several African IeDEA regions, and these publications have been cited over 10,000 times. Multiregional projects are developed in IeDEA working groups, which currently address eight clinical areas: (i) cancer, (ii) ART outcomes, (iii) hepatitis, (iv) mental health, (v) mother-infant and paediatrics, (vi) renal disease, (vii) substance use and (viii) tuberculosis. Multiregional research concepts are discussed in the Executive Committee of IeDEA, revised and approved or rejected. In recent years, several analyses were done in collaboration with WHO or UNAIDS [21–23]. The number of publications reporting multi-regional analyses from several African IeDEA regions increased over time, from one such publication in 2007 to 24 multiregional publications in 2018. Some of the key studies are summarised below, with a focus on more recent and on multiregional analyses.

For peer review only - http://bmjopen.bmj.com/site/about/guidelines.xhtml

Treatment outcomes in adults, adolescents, children and pregnant women: Several studies examined outcomes in people living with HIV-1 or HIV-2 who initiate ART, including determinants of mortality, of switching to second-line and third-line ART, drug resistance, loss to follow-up and the immunological and virological response to different ART regimens [24–37]. For example, the African IeDEA regions contributed importantly to a large-scale analysis of outcomes in adolescents living with perinatally acquired HIV, which showed that HIV-associated mortality during adolescence was substantially higher in sub-Saharan Africa, South and Southeast Asia, and South America and the Caribbean than in Europe [36]. A similar analysis of adolescents living with HIV showed that mortality and loss to follow-up were worse among those entering care at 15 years or older [37]. The authors concluded that adolescents must be evaluated separately from younger children and adults to identify population-specific reasons for death and loss to follow-up [37].

Programme-level mortality: It became clear early on during the scale-up of ART in sub-Saharan Africa, that loss to follow-up of patients initiating ART was substantial [38], and that mortality among patients lost was higher than among patients remaining in care [39]. Ignoring loss to follow-up might thus bias programme-level estimates of mortality, and much effort has gone into correcting programme-level mortality for loss to follow-up [40–46]. For example, an analysis of all four African regions showed that when analysing the uncorrected data observed in the clinics, 52% of adults and children were retained on ART, 42% were lost to follow-up and 6% had died 5 years after ART initiation [46]. After accounting for undocumented deaths and self-transfers, an estimated 67% of patients were retained on ART, 19% had stopped ART and 15% had died [46].

Co-infections and co-morbidities: IeDEA investigators have examined the prevalence and impact of co-infection with hepatitis B and C, and the epidemiology of different cancers and of (multi-drug resistant) tuberculosis, renal disease and of mental illness [8,47–58]. For example, an analysis of IeDEA data and data from the Collaboration of Observational HIV Epidemiological Research in Europe (COHERE) [59] showed that children living with HIV from sub-Saharan Africa, but not those from Europe or Asia had a high risk of developing Kaposi sarcoma after starting ART [58]. Similarly, a recent analysis of IeDEA and COHERE data showed that compared to European women, rates of cervical cancer were 11 times higher in South Africa [50]. A recent multiregional study of multi-

For peer review only - http://bmjopen.bmj.com/site/about/guidelines.xhtml

drug resistant tuberculosis included HIV positive and HIV negative adults with tuberculosis from seven high-burden countries (Côte d'Ivoire, Democratic Republic of the Congo, Kenya, Nigeria, South Africa, Peru, and Thailand). Molecular or phenotypic drug susceptibility testing was done locally and at a reference laboratory. The results showed that inaccurate local drug susceptibility testing led to under-treatment of drug-resistant tuberculosis and increased mortality [54].

The challenge of "Treat All": Nearly all countries in sub-Saharan Africa have now adopted national polices to offer ART to all PLWHIV regardless of CD4 cell count or clinical stage ('Treat All'), in order to meet the UNAIDS 90-90-90 targets. In 2011, Malawi was one of the first countries to implement such a strategy for the prevention of mother-to-child transmission (PMTCT), recommending ART for pregnant and breastfeeding women living with HIV, regardless of CD4 cell count or WHO clinical stage ("Option B+"). An IeDEA analysis of the Malawian experience showed that poor retention in care was a problem in many facilities, with early loss to follow-up particularly high in facilities with a high patient volume and in patients who start ART during pregnancy on the day of HIV diagnosis [35]. More recently, IeDEA investigators used regression discontinuity analysis to examine changes in rapid HIV treatment initiation after national "Treat All" policy adoption in six countries (Burundi, Kenya, Malawi, Rwanda, Uganda, Zambia) [60]. They showed a strong and sustained effect of the adoption of "Treat All" policies on ART initiation within 30 days of enrollment in HIV care in all six countries [60].

Future plans: At the end of 2018, IeDEA published a consensus statement [61] and a journal supplement [62] on research priorities to inform the implementation of the "Treat All" policy in children and adolescents [63], pregnant and post-partum women [64], and for mental health, substance use [65] and drug resistance [66]. These documents will guide IeDEA's future research agenda in sub-Saharan Africa. Furthermore, the creation of an IeDEA Sentinel Research Network (IeDEA-SRN) will facilitate the collection of detailed data in selected IeDEA sites on cardio-metabolic risk factors (e.g. hypertension, diabetes, dyslipidaemia) liver disease (liver fibrosis and steatosis) , mental health and substance use. In the East and Southern African regions, pharmacovigilance in pregnancy is being developed to assess the impact of ART on birth outcomes. A project involving all four African regions studies the cascade of screening for cervical cancer,

For peer review only - http://bmjopen.bmj.com/site/about/guidelines.xhtml

while the establishment of the South African HIV Cancer Match (SAM) study of over ten million PLWHIV, from linkage of national laboratory with cancer registry data, will allow the study of less common cancers. Finally, the creation of a drug resistance database as a central repository for resistance tests performed in routine clinical care is another planned addition.

COLLABORATIONS

The African regions of IeDEA have collaborated and continue to encourage collaborations with other consortia, cohort collaborations, the HIV modelling community and public health agencies as well as individuals wishing to use IeDEA data. Examples include work with COHERE [50,58,67,68], the Collaborative Initiative for Paediatric HIV Education and Research (CIPHER) [36,69] or the Measurement & Surveillance of HIV Epidemics (MeSH) consortium [22] as well as UNAIDS [22,23] and WHO [21]. Furher collaborations are welcome. Investigators wishing to work with the IeDEA data should contact the teams at the regional data centres (see www.iedea.org for contact details) and send a concept sheet for the analyses they are interested in performing and the variables that would be required. Anyone wishing to work with IeDEA must sign a data-use agreement.

For peer review only

For peer review only - http://bmjopen.bmj.com/site/about/guidelines.xhtml

FURTHER DETAILS

Acknowledgements: The authors wish to thank the clinical and administrative staff at the participating clinics. We are grateful to all PLWHIV who contributed to the IeDEA database.

Contributors: FC, PMG, and ME conceptualised the study. FC and CHDO performed statistical analyses. FC and ME wrote the first draft of the paper. CHDO, KA, AJ, EB, SB, FD, MAD, SND, KM, BSM, DN, KWK, CY, PMG and ME contributed to interpreting the data and to the writing and revising of the manuscript.

Funding: The International Epidemiology Databases to Evaluate AIDS (IeDEA) is supported by the U.S. National Institutes of Health's National Institute of Allergy and Infectious Diseases, the Eunice Kennedy Shriver National Institute of Child Health and Human Development, the National Cancer Institute, the National Institute of Mental Health, the National Institute on Drug Abuse and Alcoholism, the National Institute of Diabetes and Digestive and Kidney Diseases, the Fogarty International Center, the National Library of Medicine, and the Office of the Director: Central Africa, U01AI096299; East Africa, U01AI069911; Southern Africa, U01AI069924; West Africa, U01AI069919. Informatics resources are supported by the Harmonist project, R24AI124872. ME was supported by special project funding (Grant No. 174281) from the Swiss National Science Foundation.

Disclaimer: The contents are the responsibility of the authors and do not necessarily reflect the views of NIAD or the US Government. The funders had no role in study design, data collection and analysis, decision to publish or preparation of the manuscript.

Competing interests: None declared.

Ethics approval: The Ethics Committee of the Canton of Bern, the Ethics Committee of the University of Cape Town and the local ethics committees or institutional review boards all approved the use of routine clinical data for research within the IeDEA collaboration. For studies requiring additional data collection, separate ethics approval and study-specific informed consent is sought.

For peer review only - http://bmjopen.bmj.com/site/about/guidelines.xhtml

Provenance and peer review: Not commissioned; externally peer reviewed.

Data sharing statement: Investigators wishing to work with IeDEA data should contact the regional data centres (see www.iedea.org) and send a concept sheet for the analyses they are interested in performing and the variables that would be required. See www.iedeades.org for list of variables. Those wishing to work with the data must sign a data-use agreement.

Open Access: This is an Open Access article distributed in accordance with the Creative Commons Attribution Non Commercial (CC BY-NC 4.0) license, which permits others to distribute, remix, adapt, build upon this work non-commercially, and license their derivative works on different terms, provided the original work is properly cited and the use is non-commercial. See: http://creativecommons.org/licenses/by-nc/4.0/.

For peer review only

REFERENCES

1 UNAIDS. Global AIDS update 2016. Geneva, Switzerland: 2016.

2 UNAIDS. UNAIDS Data 2018. UNAIDS https://www.aidsdatahub.org/unaids-data-2018-unaids-2018

3 UNAIDS. 90-90-90: An ambitious treatment target to help end the AIDS epidemic. Geneva, Switzerland: 2014.

4 UNAIDS. UNAIDS data 2019. https://www.unaids.org/en/resources/documents/2019/2019-UNAIDS-data (accessed 16 Jan 2020).

5 Egger M, Ekouevi DK, Williams C, *et al.* Cohort Profile: The international epidemiological databases to evaluate AIDS (IeDEA) in sub-Saharan Africa. *Int J Epidemiol* 2012;**41**:1256–64. doi:10.1093/ije/dyr080

6 Harris PA, Taylor R, Minor BL, *et al.* The REDCap consortium: Building an international community of software platform partners. *J Biomed Inform* 2019;**95**:103208. doi:10.1016/j.jbi.2019.103208

7 Duda SNSN, Farr AMAM, Lindegren MLML, *et al.* Characteristics and comprehensiveness of adult HIV care and treatment programmes in Asia-Pacific, sub-Saharan Africa and the Americas: results of a site assessment conducted by the International epidemiologic Databases to Evaluate AIDS Collaboration. *J Int AIDS Soc* 2014;**17**:19045. doi:10.7448/IAS.17.1.19045

8 Parcesepe AM, Mugglin C, Nalugoda F, *et al.* Screening and management of mental health and substance use disorders in HIV treatment settings in low- and middle-income countries within the global IeDEA consortium. *J Int AIDS Soc* 2018;**21**:e25101. doi:10.1002/jia2.25101

9 Ballif M, Nhandu V, Wood R, *et al.* Detection and management of drug-resistant tuberculosis in HIV-infected patients in lower-income countries. *Int J Tuberc Lung Dis Off J Int Union Tuberc Lung Dis* 2014;**18**:1327–36. doi:10.5588/ijtld.14.0106

10 Fenner L, Forster M, Boulle A, *et al.* Tuberculosis in HIV programmes in lower-income countries: practices and risk factors. *Int J Tuberc Lung Dis Off J Int Union Tuberc Lung Dis* 2011;**15**:620–7. doi:10.5588/ijtld.10.0249

11 Ballif M, Renner L, Claude Dusingize J, *et al.* Tuberculosis in Pediatric Antiretroviral Therapy Programs in Low- and Middle-Income Countries: Diagnosis and Screening Practices. *J Pediatr Infect Dis Soc* 2015;**4**:30–8. doi:10.1093/jpids/piu020

12 Horner M-J, Chasimpha S, Spoerri A, *et al.* High Cancer Burden Among Antiretroviral Therapy Users in Malawi: a Record Linkage Study of Observational HIV Cohorts and Cancer Registry Data. *Clin Infect Dis* Published Online First: 17 November 2018. doi:10.1093/cid/ciy960

For peer review only - http://bmjopen.bmj.com/site/about/guidelines.xhtml

13 Dhokotera T, Bohlius J, Spoerri A, *et al.* The burden of cancers associated with HIV in the South African public health sector, 2004-2014: a record linkage study. *Infect Agent Cancer* 2019;**14**:12. doi:10.1186/s13027-019-0228-7

14 Boulle A, Schomaker M, May MT, *et al.* Mortality in patients with HIV-1 infection starting antiretroviral therapy in South Africa, Europe, or North America: a collaborative analysis of prospective studies. *PLoS Med* 2014;**11**:e1001718. doi:10.1371/journal.pmed.1001718

15 Davies M-A, Tsondai P, Tiffin N, *et al.* Where do HIV-infected adolescents go after transfer? - Tracking transition/transfer of HIV-infected adolescents using linkage of cohort data to a health information system platform. *J Int AIDS Soc* 2017;**20**:21668. doi:10.7448/IAS.20.4.21668

16 WHO. Consolidated guidelines on the use of antiretroviral drugs for treating and preventing HIV infection: recommendations for a public health approach. Geneva, Switzerland: 2013.

17 (PEPFAR). PEPFAR 2018 Country Operational Plan Guidance for Standard Process Countries Table of Contents. 2018.

18 Cottrell ML, Hadzic T, Kashuba ADM. Clinical Pharmacokinetic, Pharmacodynamic and Drug-Interaction Profile of the Integrase Inhibitor Dolutegravir. *Clin Pharmacokinet* 2013;**52**:981–94. doi:10.1007/s40262-013-0093-2

19 Mesplede T, Quashie PK, Osman N, *et al.* Viral fitness cost prevents HIV-1 from evading dolutegravir drug pressure. *Retrovirology* 2013;**10**:22. doi:10.1186/1742-4690-10-22

20 Zash R, Holmes L, Diseko M, *et al.* Neural-Tube Defects and Antiretroviral Treatment Regimens in Botswana. *N Engl J Med* 2019;:NEJMoa1905230. doi:10.1056/NEJMoa1905230

21 Zaniewski E, Tymejczyk O, Kariminia A, *et al.* IeDEA-WHO Research-Policy Collaboration: contributing real-world evidence to HIV progress reporting and guideline development. *J Virus Erad* 2018;**4**:9–15.

22 Anderegg N, Johnson LF, Zaniewski E, *et al.* All-cause mortality in HIV-positive adults starting combination antiretroviral therapy: correcting for loss to follow-up. *AIDS Lond Engl* 2017;**31 Suppl 1**:S31–40. doi:10.1097/QAD.0000000000001321

23 Mahy M, Penazzato M, Ciaranello A, *et al.* Improving estimates of children living with HIV from the Spectrum AIDS Impact Model. *AIDS Lond Engl* 2017;**31 Suppl 1**:S13–22. doi:10.1097/QAD.0000000000001306

24 Davies M-AM-AA, May M, Bolton-Moore C, *et al.* Prognosis of Children With HIV-1 Infection Starting Antiretroviral Therapy in Southern Africa. *Pediatr Infect Dis J* 2014;**33**:608–16. doi:10.1097/INF.0000000000000214

25 Petersen ML, Tran L, Geng EH, *et al.* Delayed switch of antiretroviral therapy after virologic failure associated with elevated mortality among HIV-infected adults in Africa. *AIDS Lond Engl* 2014;**28**:2097–107. doi:10.1097/QAD.0000000000000349

For peer review only - http://bmjopen.bmj.com/site/about/guidelines.xhtml

26 Tchounga BK, Hønge BL, Eholie SP, *et al.* Effect of sex and age on outcomes among HIV-2-infected patients starting antiretroviral therapy in West Africa. *AIDS Lond Engl* 2016;**30**:2707–14. doi:10.1097/QAD.0000000000001232

27 Giles ML, Achhra AC, Abraham AG, *et al.* Sex-based differences in antiretroviral therapy initiation, switching and treatment interruptions: global overview from the International Epidemiologic Databases to Evaluate AIDS (IeDEA). *J Int AIDS Soc* 2018;**21**:e25149. doi:10.1002/jia2.25149

28 Haas AD, Keiser O, Balestre E, *et al.* Monitoring and switching of first-line antiretroviral therapy in adult treatment cohorts in sub-Saharan Africa: Collaborative analysis. *Lancet HIV* 2015;**2**:e271–e278. doi:10.1016/S2352-3018(15)00087-9

29 Chimbetete C, Katzenstein D, Shamu T, *et al.* HIV-1 Drug Resistance and Third-Line Therapy Outcomes in Patients Failing Second-Line Therapy in Zimbabwe. *Open Forum Infect Dis* 2018;**5**:1–8. doi:10.1093/ofid/ofy005

30 Wools-Kaloustian K, Marete I, Ayaya S, *et al.* Time to First-Line ART Failure and Time to Second-Line ART Switch in the IeDEA Pediatric Cohort. *Jaids-J Acquir Immune Defic Syndr* 2018;**78**:221–30. doi:10.1097/QAI.0000000000001667

31 Charpentier C, Eholié S, Anglaret X, *et al.* Genotypic resistance profiles of HIV-2-treated patients in West Africa. *AIDS Lond Engl* 2014;**28**:1161–9. doi:10.1097/QAD.0000000000000244

32 Kiragga AN, Lok JJ, Musick BS, *et al.* CD4 trajectory adjusting for dropout among HIV-positive patients receiving combination antiretroviral therapy in an East African HIV care centre. *J Int AIDS Soc* 2014;**17**:18957. doi:10.7448/IAS.17.1.18957

33 Geng EH, Neilands TB, Thièbaut R, *et al.* CD41 T cell recovery during suppression of HIV replication: an international comparison of the immunological efficacy of antiretroviral therapy in North America, Asia and Africa. *Int J Epidemiol* 2015;**44**:251–63. doi:10.1093/ije/dyu271

34 Technau K-G, Schomaker M, Kuhn L, *et al.* Virologic response in children treated with abacavir-compared with stavudine-based antiretroviral treatment: a South African multi-cohort analysis. *Pediatr Infect Dis J* 2014;**33**:617–22. doi:10.1097/INF.0000000000000222

35 Tenthani L, Haas AD, Tweya H, *et al.* Retention in care under universal antiretroviral therapy for HIV-infected pregnant and breastfeeding women ('Option B+') in Malawi. *AIDS* 2014;**28**:589–598 Keywords:

36 Collaborative Initiative for Paediatric HIV Education and Research (CIPHER) Global Cohort Collaboration, Slogrove AL, Schomaker M, *et al.* The epidemiology of adolescents living with perinatally acquired HIV: A cross-region global cohort analysis. *PLoS Med* 2018;**15**:e1002514. doi:10.1371/journal.pmed.1002514

For peer review only - http://bmjopen.bmj.com/site/about/guidelines.xhtml

37 Kariminia A, Law M, Davies M, *et al.* Mortality and losses to follow-up among adolescents living with HIV in the Ie DEA global cohort collaboration. *J Int AIDS Soc* 2018;**21**:e25215. doi:10.1002/jia2.25215

38 Rosen S, Fox MP, Gill CJ. Patient retention in antiretroviral therapy programs in sub-Saharan Africa: a systematic review. *PLoSMed* 2007;**4**:e298.

39 Brinkhof MWGWMWG, Pujades-Rodriguez M, Egger M. Mortality of patients lost to follow-up in antiretroviral treatment programmes in resource-limited settings: systematic review and meta-analysis. *PLoS One* 2009;**4**:e5790. doi:10.1371/journal.pone.0005790

40 Geng EH, Odeny TA, Lyamuya RE, *et al.* Estimation of mortality among HIV-infected people on antiretroviral treatment in east Africa: a sampling based approach in an observational, multisite, cohort study. *Lancet HIV* 2015;**2**:e107–16. doi:10.1016/S2352-3018(15)00002-8

41 An M-W, Frangakis CE, Yiannoutsos CT. Choosing profile double-sampling designs for survival estimation with application to President's Emergency Plan for AIDS Relief evaluation. *Stat Med* 2014;**33**:2017–29. doi:10.1002/sim.6087

42 Schomaker M, Gsponer T, Estill J, *et al.* Non-ignorable loss to follow-up: correcting mortality estimates based on additional outcome ascertainment. *Stat Med* 2014;**33**:129–142. doi:10.1002/sim.5912

43 Brinkhof MWG, Spycher BD, Yiannoutsos C, *et al.* Adjusting mortality for loss to follow-up: Analysis of five art programmes in sub-saharan africa. *PLoS ONE* 2010;**5**:3–8. doi:10.1371/journal.pone.0014149

44 Egger M, Spycher BD, Sidle J, *et al.* Correcting mortality for loss to follow-up: a nomogram applied to antiretroviral treatment programmes in sub-Saharan Africa. *PLoS Med* 2011;**8**:e1000390. doi:10.1371/journal.pmed.1000390

45 Yiannoutsos CT, Johnson LF, Boulle A, *et al.* Estimated mortality of adult HIV-infected patients starting treatment with combination antiretroviral therapy. *Sex Transm Infect* 2012;**88 Suppl 2**:i33—-43.

46 Haas AD, Zaniewski E, Anderegg N, *et al.* Retention and mortality on antiretroviral therapy in sub-Saharan Africa: collaborative analyses of HIV treatment programmes. *J Int AIDS Soc* 2018;**21**:e25084. doi:10.1002/jia2.25084

47 Hector J, Vinikoor M, Chilengi R, *et al.* No Impact of Hepatitis B Virus Infection on Early Mortality Among Human Immunodeficiency Virus–Infected Patients in Southern Africa. *Clin Infect Dis* 2018;**67**:1310–1311. doi:10.1093/cid/ciy304

48 Wandeler G, Mulenga L, Hobbins M, *et al.* Absence of Active Hepatitis C Virus Infection in Human Immunodeficiency Virus Clinics in Zambia and Mozambique. *Open Forum Infect Dis* 2016;**3**:ofw049. doi:10.1093/ofid/ofw049

For peer review only - http://bmjopen.bmj.com/site/about/guidelines.xhtml

49 Jaquet A, Wandeler G, Nouaman M, *et al.* Alcohol use, viral hepatitis and liver fibrosis among HIV-positive persons in West Africa: A cross-sectional study. *J Int AIDS Soc* 2017;**20**:21424. doi:10.7448/IAS.20.1.21424

50 Rohner E, Bütikofer L, Schmidlin K, *et al.* Cervical cancer risk in women living with HIV across four continents: A multicohort study: Regional cervical cancer risk in HIV-positive women. *Int J Cancer* Published Online First: 19 June 2019. doi:10.1002/ijc.32260

51 Brown SA, Abbas S, Davies M-A, *et al.* Brief Report: Pediatric Cancer Burden and Treatment Resources Within the Pediatric IeDEA Consortium. *J Acquir Immune Defic Syndr 1999* 2017;**76**:60–4. doi:10.1097/QAI.0000000000001453

52 Semeere A, Wenger M, Busakhala N, *et al.* A prospective ascertainment of cancer incidence in sub-Saharan Africa: The case of Kaposi sarcoma. *Cancer Med* 2016;**5**:914–28. doi:10.1002/cam4.618

53 Carlucci JG, Blevins Peratikos M, Kipp AM, *et al.* Tuberculosis Treatment Outcomes Among HIV/TB-Coinfected Children in the International Epidemiology Databases to Evaluate AIDS (IeDEA) Network. *J Acquir Immune Defic Syndr 1999* 2017;**75**:156–63. doi:10.1097/QAI.0000000000001335

54 Zurcher K, Ballif M, Fenner L, *et al.* Drug susceptibility testing and mortality in patients treated for tuberculosis in high-burden countries: a multicentre cohort study. *Lancet Infect Dis* 2019;**19**:298–307. doi:10.1016/S1473-3099(18)30673-X

55 Gygli SM, Keller PM, Ballif M, *et al.* Whole-Genome Sequencing for Drug Resistance Profile Prediction in Mycobacterium tuberculosis. *Antimicrob Agents Chemother* 2019;**63**:e02175-18. doi:10.1128/AAC.02175-18

56 Zürcher K, Ballif M, Kiertiburanakul S, *et al.* Diagnosis and clinical outcomes of extrapulmonary tuberculosis in antiretroviral therapy programmes in low- and middle-income countries: a multicohort study. *J Int AIDS Soc* 2019;**22**:e25392. doi:10.1002/jia2.25392

57 Mulenga L, Musonda P, Mwango A, *et al.* Effect of baseline renal function on tenofovir-containing antiretroviral therapy outcomes in Zambia. *Clin Infect Dis Off Publ Infect Dis Soc Am* 2014;**58**:1473–80. doi:10.1093/cid/ciu117

58 Rohner E, Schmidlin K, Zwahlen M, *et al.* Kaposi Sarcoma Risk in HIV-Infected Children and Adolescents on Combination Antiretroviral Therapy From Sub-Saharan Africa, Europe, and Asia. *Clin Infect Dis* 2016;**63**:ciw519. doi:10.1093/cid/ciw519

59 Chêne G, Phillips A, Costagliola D, *et al.* Cohort profile: Collaboration of Observational HIV Epidemiological Research Europe (COHERE) in EuroCoord. *Int J Epidemiol* 2017;**46**:797. doi:10.1093/ije/dyw211

60 Tymejczyk O, Brazier E, Yiannoutsos CT, *et al.* Changes in rapid HIV treatment initiation after national “treat all” policy adoption in 6 sub-Saharan African countries: Regression discontinuity analysis. *PLOS Med* 2019;**16**:e1002822. doi:10.1371/journal.pmed.1002822

For peer review only

For peer review only - http://bmjopen.bmj.com/site/about/guidelines.xhtml

61 Yotebieng M, Brazier E, Addison D, *et al.* Research priorities to inform “Treat All” policy implementation for people living with HIV in sub-Saharan Africa: a consensus statement from the International epidemiology Databases to Evaluate AIDS (Ie DEA). *J Int AIDS Soc* 2019;**22**:e25218. doi:10.1002/jia2.25218

62 Nash D, Yotebieng M, Sohn AH. Treating all people living with HIV in sub-Saharan Africa: a new era calling for new approaches. *J Virus Erad* 2018;**4**:1–4.

63 Enane LA, Davies M-A, Leroy V, *et al.* Traversing the cascade: urgent research priorities for implementing the “treat all” strategy for children and adolescents living with HIV in sub-Saharan Africa. *J Virus Erad* 2018;**4**:40–46.

64 Abuogi LL, Humphrey JM, Mpody C, *et al.* Achieving UNAIDS 90-90-90 targets for pregnant and postpartum women in sub-Saharan Africa: progress, gaps and research needs. *J Virus Erad* 2018;**4**:33–9.

65 Lancaster KE, Hetrick A, Jaquet A, *et al.* Substance use and universal access to HIV testing and treatment in sub-Saharan Africa: implications and research priorities. *J Virus Erad* 2018;**4**:26–32.

66 de Waal R, Lessells R, Hauser A, *et al.* HIV drug resistance in sub-Saharan Africa: public health questions and the potential role of real-world data and mathematical modelling. *J Virus Erad* 2018;**4**:55–8.

67 Panayidou K, Davies M-A, Anderegg N, *et al.* Global temporal changes in the proportion of children with advanced disease at the start of combination antiretroviral therapy in an era of changing criteria for treatment initiation. *J Int AIDS Soc* 2018;**21**:e25200. doi:10.1002/jia2.25200

68 Anderegg N, Panayidou K, Abo Y, *et al.* Global Trends in CD4 Cell Count at the Start of Antiretroviral Therapy: Collaborative Study of Treatment Programs. *Clin Infect Dis* 2018;**66**:893–903. doi:10.1093/cid/cix915

69 Collaborative Initiative for Paediatric HIV Education and Research (CIPHER) Global Cohort Collaboration. Incidence of switching to second-line antiretroviral therapy and associated factors in children with HIV: an international cohort collaboration. *Lancet HIV* 2019;**6**:e105–15. doi:10.1016/S2352-3018(18)30319-9

For peer review only

For peer review only - http://bmjopen.bmj.com/site/about/guidelines.xhtml

Table 1: Characteristics of 240 facilities providing ART in the African regions if the IeDEA (source: site assessment survey 2017).

	West Africa	Central Africa	East Africa	Southern Africa	All regions (%)
**No of active facilities**	17	19	72	132	240
**No of patients on ART**	45,015	32,754	252,266	1,066,591	1,396,626
**Location**					
Urban	17	19	19	65	120 (50)
Rural	0	0	52	67	119 (50)
Missing	0	0	1	0	1 (0)
**Level of care**					
Primary	4	12	49	107	172 (72)
Secondary	1	0	16	20	37 (15)
Tertiary	12	7	6	5	30 (13)
Missing	0	0	1	0	1 (0)
**Type of facility**					
Public	12	16	69	128	225 (94)
Private	2	3	3	4	12 (5)
Missing	3	0	0	0	3 (1)
**Viral load**					
Routine testing	12	16	46	74	148 (62)
Tests performed onsite	8	6	4	3	21 (9)
Tests performed offsite	6	13	53	127	199 (83)
**CD4 monitoring**					
Routine testing	14	6	9	97	126 (53)
Tests performed onsite	11	8	23	51	93 (39)
Tests performed offsite	3	11	35	71	120 (50)
**HIV-1 genotypic drug resistance**					
Routine testing	2	2	10	48	62 (26)
**Routine tracing of patients LTFU**					
Yes	13	19	56	93	181 (75)
No	1	0	1	37	39 (16)
Missing	3	0	15	2	20 (8)
**Tracing method***					
Phone	14	18	57	110	199 (83)
SMS/mail/email	2	2	10	14	28 (15)
Home visit	9	17	52	129	207 (86)
**Medication disruption/stock outs over last 12 months**					
First-line ART	5	2	20	32	59 (25)
Second-line ART	5	6	18	16	45 (19)

*Sites may use more than 1 method.

For peer review only - http://bmjopen.bmj.com/site/about/guidelines.xhtml

Table 2: Proportion of patients with different nucleoside and non-nucleoside reverse transcriptase inhibitor regimens at the start of first-line antiretroviral therapy, by time period and region.

	Central Africa								East Africa							
	2001-2002	2003-2004	2005-2006	2007-2008	2009-2010	2011-2012	2013-2014	2015-2016	2001-2002	2003-2004	2005-2006	2007-2008	2009-2010	2011-2012	2013-2014	2015-2016
No of patients	-	927	2,766	4,930	4,848	5,574	6,048	5,423	819	6,710	27,614	33,290	39,674	46,578	44,834	38,905
**NRTI**																
FTC+TDF	-	1.2	4.4	0.8	3.5	5.0	6.6	9.2	0.8	5.4	0.3	1.0	0.9	1.1	1.4	0.7
3TC+TDF	-	6.4	4.1	3.9	34.4	49.7	60.1	76.5	2.7	5.0	1.1	2.3	9.3	55.1	77.5	89.7
3TC+D4T	-	60.0	55.6	33.7	8.8	2.7	0.1	0.0	78.9	80.0	83.2	54.1	37.4	3.0	0.5	0.1
3TC+AZT	-	29.6	32.7	54.9	48.6	35.9	27.0	6.6	14.0	9.0	14.8	41.8	46.4	35.3	16.6	6.4
3TC+ABC	-	1.1	1.4	2.1	2.8	4.1	5.1	7.3	0.6	0.3	0.4	0.8	5.9	5.4	4.0	3.2
Other	-	1.8	1.8	4.7	1.9	2.6	1.1	0.4	3.1	0.4	0.2	0.1	0.2	0.1	0.0	0.0
**NNRTI**																
NVP	-	64.1	65	73.6	66.6	49.2	22.5	6.3	73.1	82.6	80.9	76.4	72.2	50.4	21.3	8.1
EFV	-	35.7	34.3	25.8	32.9	50.1	76.9	93.4	24.8	15.4	17.7	22.6	27.2	49.3	78.4	91.5
Other	-	0.2	0.7	0.7	0.5	0.7	0.6	0.2	2.0	2.0	1.4	1.0	0.6	0.3	0.4	0.4

	Southern Africa								West Africa							
	2001-2002	2003-2004	2005-2006	2007-2008	2009-2010	2011-2012	2013-2014	2015-2016	2001-2002	2003-2004	2005-2006	2007-2008	2009-2010	2011-2012	2013-2014	2015-2016
No of patients	1,345	19,434	86,775	122,953	146,425	161,014	182,330	179,424	822	3533	6,348	6,490	7,748	6,836	6,136	4,451
**NRTI**																
FTC+TDF	2.0	0.5	0.5	17.4	24.9	34.4	62.2	51.9	0.6	0.0	0.1	1.5	11.5	17.6	11.5	11.6
3TC+TDF	2.5	1.1	1.1	2.1	17.1	33.5	22.2	40.3	0.0	0.0	0.2	0.6	3.5	10.3	32.1	55.3
3TC+D4T	37.2	47.7	55.3	49.5	36.5	15.3	3.4	0.2	39.2	46.8	51.6	42.6	21.6	4.8	0.6	0.0
3TC+AZT	47.0	39.7	27.7	16.2	10.7	8.7	6.8	2.5	35.7	39.7	43.3	51.0	46.2	53.4	49.3	22.5
3TC+ABC	0.7	0.2	0.3	1.2	5.0	6.0	4.9	5.0	0.2	0.1	0.4	0.9	0.9	4.1	5.8	10.3
Other	10.6	10.9	15.1	13.6	5.8	2.2	0.5	0.1	24.3	13.4	4.5	3.4	16.3	9.8	0.6	0.3
**NNRTI**																
NVP	51.1	58.1	64.8	55.4	47.2	36.7	15.6	3.7	10.1	12.0	40.9	46.1	50.2	47.0	36.1	15.0
EFV	43.7	37.8	31.4	41.5	50.0	61.2	82.4	93.9	50.1	60.6	46.7	44.4	39.7	41.5	53.8	70.7
Other	5.2	4.1	3.7	3.1	2.9	2.1	1.9	2.4	39.8	27.4	12.5	9.6	10.2	11.5	10.1	14.3

NRTI, nucleoside reverse transcriptase inhibitor; NNRTI, non-nucleoside reverse transcriptase inhibitor

For peer review only

Legends to Figures

Figure 1: Map of the 240 active facilities participating in the four African regions of the International epidemiology Databases to Evaluate AIDS (A), together with cumulative numbers of patients starting antiretroviral therapy (B).

Figure 2: Daily number of CD4 cell counts and viral load measurements over time (bar chart) and the number of patients in care (red line).

Figure 3. Trends in mortality (A) and loss to follow (B), 2001 to 2016.

For peer review only

For peer review only - http://bmjopen.bmj.com/site/about/guidelines.xhtml

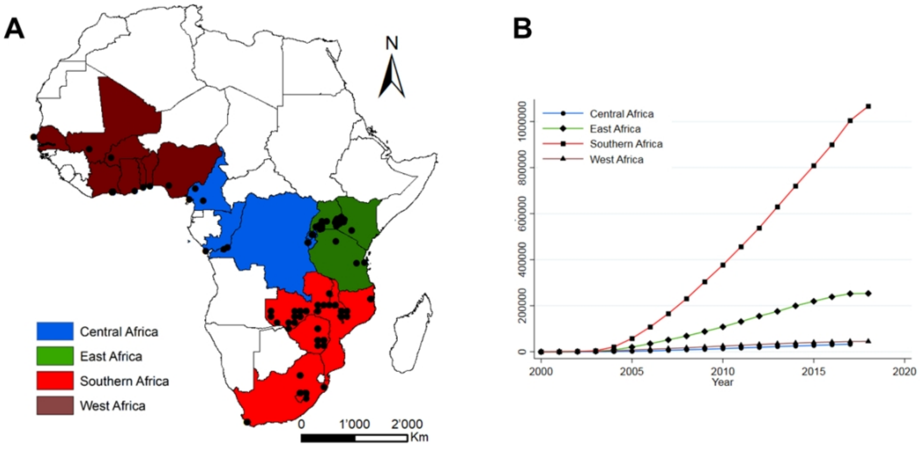

Figure 1: Map of the 240 active facilities participating in the four African regions of the International epidemiology Databases to Evaluate AIDS (A), together with cumulative numbers of patients starting antiretroviral therapy (B).

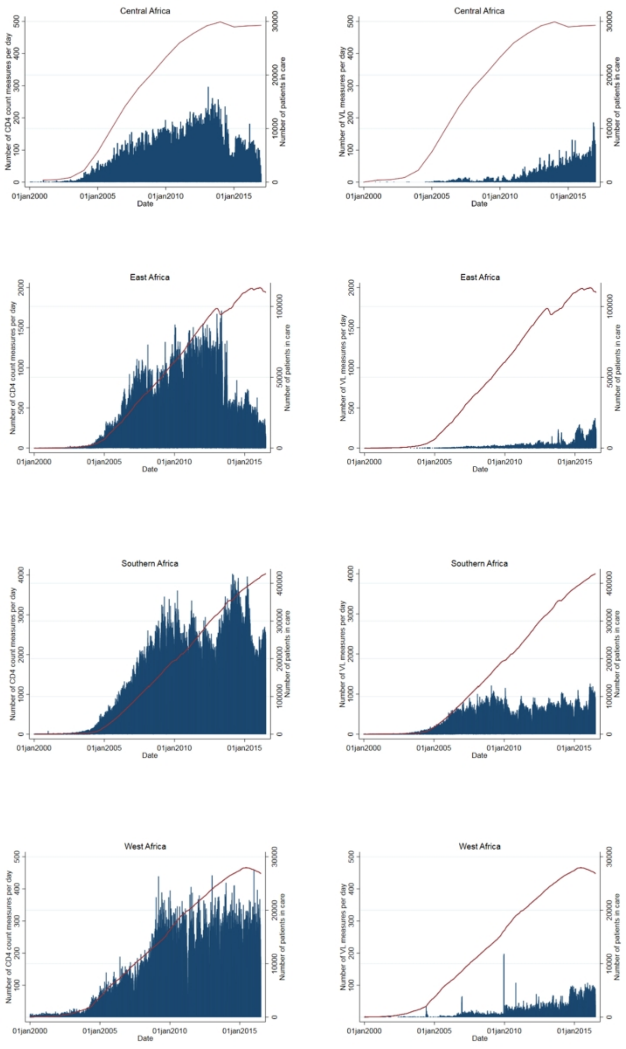

Figure 2. Daily number of CD4 cell counts and viral load measurements over time (bar chart) and the number of patients in care (red line).

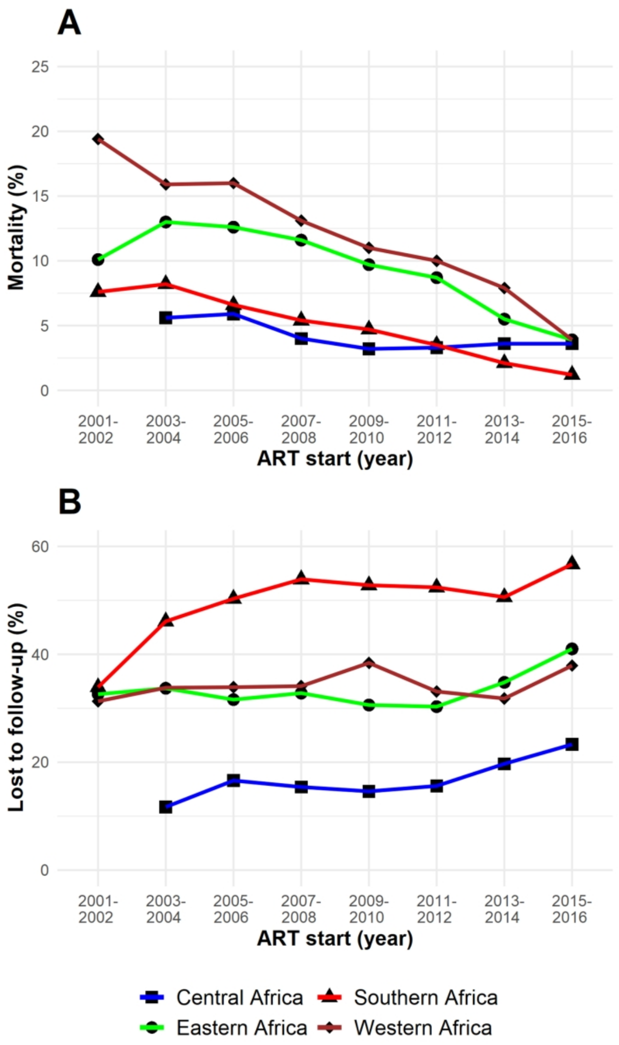

Figure 3. Trends in mortality (A) and loss to follow (B), 2001 to 2016.
